# Phosphorylated HP1α–Nucleosome Interactions in Phase Separated Environments

**DOI:** 10.1021/jacs.3c06481

**Published:** 2023-10-23

**Authors:** Nesreen Elathram, Bryce E. Ackermann, Evan T. Clark, Shelby R. Dunn, Galia T. Debelouchina

**Affiliations:** Department of Chemistry and Biochemistry, University of California, San Diego, La Jolla, California 92093, United States

## Abstract

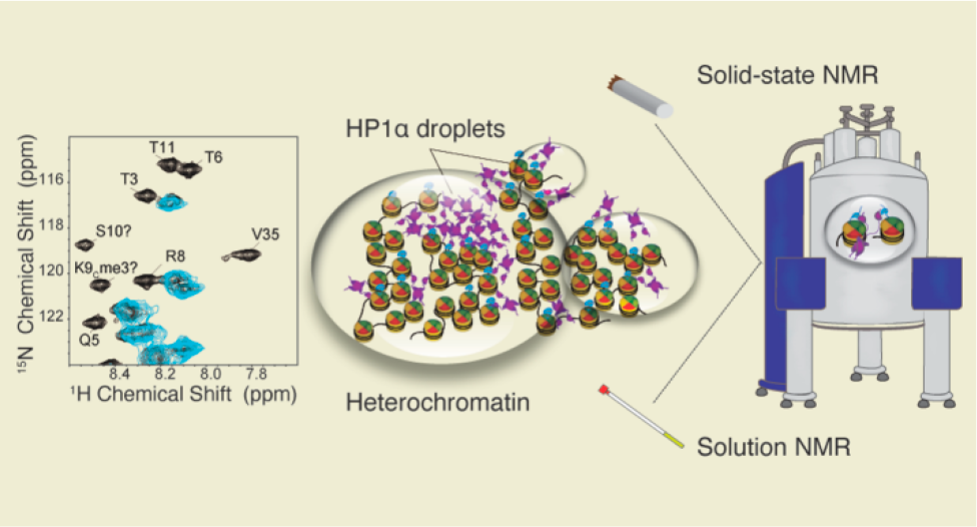

In the nucleus, transcriptionally silent genes are sequestered into heterochromatin compartments comprising nucleosomes decorated with histone H3 Lys9 trimethylation and a protein called HP1α. This protein can form liquid–liquid droplets *in vitro* and potentially organize heterochromatin through a phase separation mechanism that is promoted by phosphorylation. Elucidating the molecular interactions that drive HP1α phase separation and its consequences on nucleosome structure and dynamics has been challenging due to the viscous and heterogeneous nature of such assemblies. Here, we tackle this problem by a combination of solution and solid-state NMR spectroscopy, which allows us to dissect the interactions of phosphorylated HP1α with nucleosomes in the context of phase separation. Our experiments indicate that phosphorylated human HP1α does not cause any major rearrangements to the nucleosome core, in contrast to the yeast homologue Swi6. Instead, HP1α interacts specifically with the methylated H3 tails and slows the dynamics of the H4 tails. Our results shed light on how phosphorylated HP1α proteins may regulate the heterochromatin landscape, while our approach provides an atomic resolution view of a heterogeneous and dynamic biological system regulated by a complex network of interactions and post-translational modifications.

## Introduction

On a molecular level, the eukaryotic genome is packaged as folded nucleosome units that consist of 147 base pairs of DNA wrapped around an octamer core of histone proteins.^1^ These proteins, two copies each of histone H3, H4, H2A, and H2B, form a relatively rigid α-helical core, while the flexible histone tails extend away from the nucleosome surface and participate in nucleosome–nucleosome and nucleosome–protein interactions.^2,3^ The nucleosome and the histone tails, in particular, are subject to numerous post-translational modifications (PTMs) that play a key role in nucleosome dynamics and recognition.^4,5^ On a global nuclear level, the eukaryotic genome is organized into functional domains that contain actively transcribed genes (euchromatin) or transcriptionally silent genes (heterochromatin). Each of these domains is characterized with a specific pattern of histone PTMs and interacting proteins that modulate the chromatin environment and mediate gene activation or repression.^6^

One of the main components in heterochromatin environments is a protein called heterochromatin protein 1α (HP1α). This 191-residue protein binds to chromatin regions enriched in histone H3 lysine 9 trimethylation (H3 K9me3), where it can further recruit methyltransferases and help promote heterochromatin spreading.^7^ HP1α consists of two folded domains and three intrinsically disordered regions (Figure S1), and it typically functions in the form of a dimer with a *K*_d_ of <1 μM.^8^ The first folded domain is a chromodomain (CD) that recognizes and binds to H3 K9me3, while the second folded chromoshadow domain (CSD) is responsible for dimerization and interactions with other proteins.^9−11^ The dynamic N-terminus facilitates interactions with other HP1α dimers, while the disordered hinge region can interact with DNA.^8,12,13^ HP1α dimers bind methylated H3 K9me3 and can bridge nucleosomes that are close either in sequence or in space.^14−16^ Until recently, the prevailing view was that the resulting compaction would prevent transcription factors and other activating proteins from access to DNA, resulting in gene silencing.^15^ The recent discovery of the phase separation properties of HP1α, however, has added more complexity to this view.^8,15,17−19^ In the phase separation model, HP1α clusters around chromatin domains enriched in H3 K9me3 and engulfs them into liquid droplets that selectively exclude activating proteins.^8,15,17,20,21^ This behavior is mediated by N-terminal phosphorylation and interactions with DNA.^8,12,13^ In certain cases, e.g., *Drosophila* embryo development, maturation into gel-like states has been observed, presumably to stabilize heterochromatin domains over time.^17^

In a previous study, we used MAS NMR spectroscopy to follow the maturation of HP1α liquid droplets to a gel state and discovered that chromatin can significantly slow down this process.^22^ We also observed that gelation affects the dynamics of specific serine residues on HP1α. In this study, we take the chromatin point of view and evaluate how HP1α binding and phase separation influence the nucleosome structure and dynamics. While it is clear that HP1α interacts with the methylated H3 tail through its CD domain,^9,10^ previous studies disagree as to the extent of interaction between HP1α and the nucleosome core.^23−28^ For example, a cryo-EM structural model indicated that the HP1α dimer can link neighboring nucleosomes without an extensive interaction with either core.^23^ Unfortunately, the low resolution of this model precluded the observation of any potential local perturbations of the nucleosome structure in the presence of HP1α. In contrast, biochemical and solution NMR studies have proposed that HP1α can pry open the DNA–histone interface and interact with the αN helix of H3.^25,26^ This segment of H3 contains a PXXVXL motif that is similar to the PXVXL motif found in proteins that bind to the HP1α CSD dimer interface.^29^ These studies, however, were not performed in the context of intact methylated nucleosomes. To further complicate the matter, different HP1 variants appear to interact with nucleosomes to different extents. For example, solution NMR experiments have shown that HP1β, a mammalian paralogue, interacts with methylated nucleosomes only through the methylated H3 tail.^24^ On the other hand, the fission yeast homologue Swi6 leads to substantial reorganization of the nucleosome core, which has been implicated as an important factor in the phase separation mechanism of heterochromatin domains.^27^

As the interactions of HP1α with intact methylated nucleosomes have not yet been characterized at atomic resolution, here we set out to fill this gap with a combination of solution and solid-state NMR experiments. This approach enables the characterization of both flexible and rigid protein components to gain a comprehensive molecular view of the complex HP1α–nucleosome system. In addition, solid-state magic angle spinning (MAS) NMR spectroscopy is ideally suited for the analysis of viscous heterogeneous environments such as liquid droplets and gels,^22,30,31^ thus providing the opportunity to understand how these environments shape nucleosome structure and dynamics. Using these spectroscopic tools, we dissect the interactions of the CD domain and the CSD dimer with the nucleosome and capture the effect of HP1α phase separation on the nucleosome core and tails. We focus on the interactions between nucleosomes and HP1α phosphorylated at its NTE as this post-translational modification is constitutively present in cells and is a major driving force for phase separation *in vitro*.^8,13,32,33^ Our results indicate that phosphorylated HP1α primarily contacts the nucleosome through a CD–H3 K9me3 interaction without major rearrangements of the nucleosome core.

## Results

### pHP1α Interacts with the T3–T11 Region of H3 in Methylated Nucleosomes

**Figure 1:**
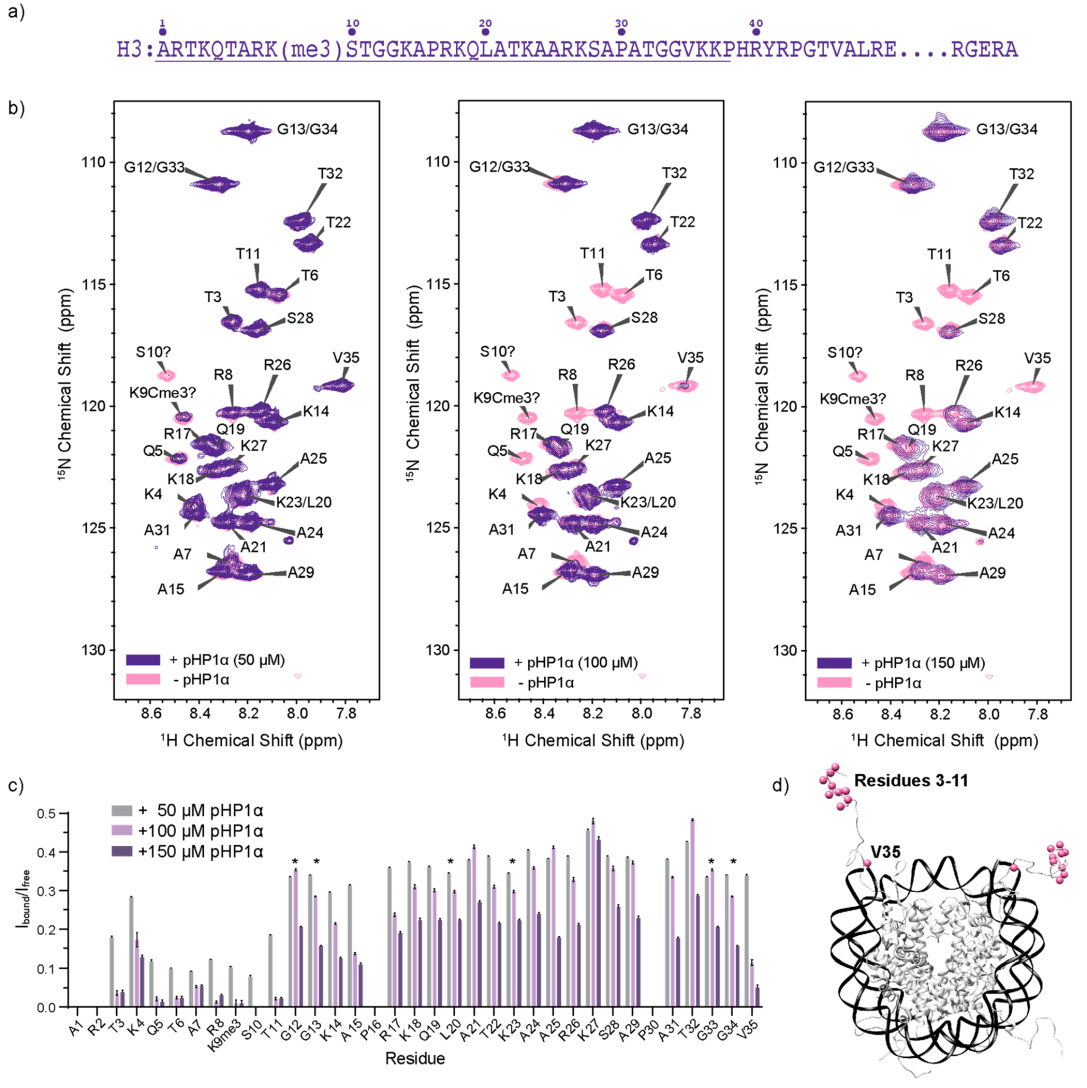
Solution NMR spectroscopy of pHP1α–H3 tail interactions. (a) H3 sequence where the underlined residues mark the dynamic N-terminal tail. (b) ^1^H–^15^N HSQC experiments of 150 μM ^13^C,^15^N–H3 labeled mononucleosomes with increasing concentrations of pHP1α. (c) Analysis of the peak intensities in the spectra shown in part b. The asterisks denote overlapped peaks where analysis for the individual residue could not be performed, i.e., G13/G34, G12/G33, and L20/K23. Note that the peak tentatively assigned to K9Cme3 is new, while the tentative S10 peak appears to be shifted compared to its position in HSQC spectra of wild-type nucleosomes.^36^ Error bars are calculated based on the signal-to-noise for each cross-peak. (d) H3 tail residues that experience significant changes in intensity upon pHP1α binding.

All HP1 proteins interact with H3 K9 methylated histone tails through the CD domain.^9,10^ While this interaction is well characterized, most structural studies have been performed in the context of an excised CD domain and H3 tail peptides rather than full-length HP1 proteins and nucleosomes.^34,35^ To understand the effect of full-length HP1α on the dynamics of the H3 tail (Figure 1a) in the nucleosome context, we started with solution NMR spectroscopy. While nucleosomes are too large to be observed in full, the histone tails experience fast rotational correlation motions that make them visible in HSQC experiments without deuteration. In the case of H3, residues 1–36 yield strong and well resolved signals.^36^ We therefore prepared ^13^C,^15^N–H3 labeled mononucleosomes that contain a lysine trimethylation mimic at position 9 of the sequence (Figures S2 and S3).^37^ Mono-, di-, and trimethylation lysine mimics have been extensively used in the biophysical and structural characterization of nucleosome interactions,^14,37,38^ although it is important to note that they may result in slightly weaker binding between the H3 tail and the CD domain (e.g., 1 μM vs 10 μM in peptide binding studies).^14^

We then added increasing amounts of full-length HP1α and followed the H3 peak intensities in ^1^H–^15^N HSQC experiments (Figure 1b). Throughout this study, we used natural abundance HP1α that is phosphorylated at residues S11–14 on the NTE (pHP1α). We prepared this construct through dual expression in *E. coli* with casein kinase II (CKII), which results in essentially complete phosphorylation of the four serine residues with minimal phosphorylation elsewhere in the protein (Figure S4). Previous literature suggests that pHP1α has higher specificity toward H3K9me3 nucleosomes due to diminished binding to DNA and improved recognition of the methylation mark.^33^ Since phase separation conditions can affect the quality of the solution NMR spectra, we used substoichiometric ratios of pHP1α to mononucleosomes, which resulted in clear samples without droplets (Figure S5) and yielded well resolved assignable spectra (Figure 1b). We also note that we performed all NMR structural studies (both solution and solid state) under low salt conditions to compare to previous work and to take advantage of published assignments.^39−43^

As the concentration of pHP1α increased, there was a global reduction in the HSQC intensity for all observed peaks in the H3 tail. Some sites, however, experienced more severe changes (Figure 1c). This included the cross-peaks for residues 3–11 that are centered around the H3 K9me3 binding site (Figure 1d). The crystal structure of the CD domain with an H3 peptide indicates direct binding interactions for residues 5–10.^34^ Our detected binding region is slightly larger, possibly due to decreased dynamics of the additional residues upon pHP1α binding or the formation of transient interactions that are not detected in the crystal structure but are suggested by molecular dynamics simulations.^44^ More surprisingly, however, we also detected a significant change for V35, a residue that is far from the binding site but close to the DNA–histone interface. Similar experiments performed with the CD domain indicated that this domain alone is not sufficient to cause the prominent decrease in V35 peak intensity (Figure S6). Thus, other segments of the full-length pHP1α protein appear to affect residues at the DNA–histone interface through either direct interactions or propagation of dynamic changes from the H3 tail to contact points with DNA.

### The CSD Dimer Does Not Interact with Intact Nucleosomes

Having recapitulated the interactions of the pHP1α CD domain with the modified H3 tail in the context of nucleosomes, we then turned our attention to the CSD dimer. The dimerization of the CSD domains of two HP1α monomers leads to the formation of a new β-sheet surface that can recognize and bind to a PXVXL motif present in many HP1α interaction partners. This interaction is somewhat promiscuous and can also accommodate other PXVXL-like motifs.^45^ Interestingly, the αN helix of H3 contains the sequence PGTVAL, which previously prompted the hypothesis that HP1α may be able to bind this region on the nucleosome if it can gain access to it.^25,26^ This hypothesis was supported by binding studies which showed that the CSD dimer can bind an H3 peptide containing the PGTVAL sequence (with a *K*_d_ of ∼58 μM) as well as free histone proteins.^25,26^

**Figure 2:**
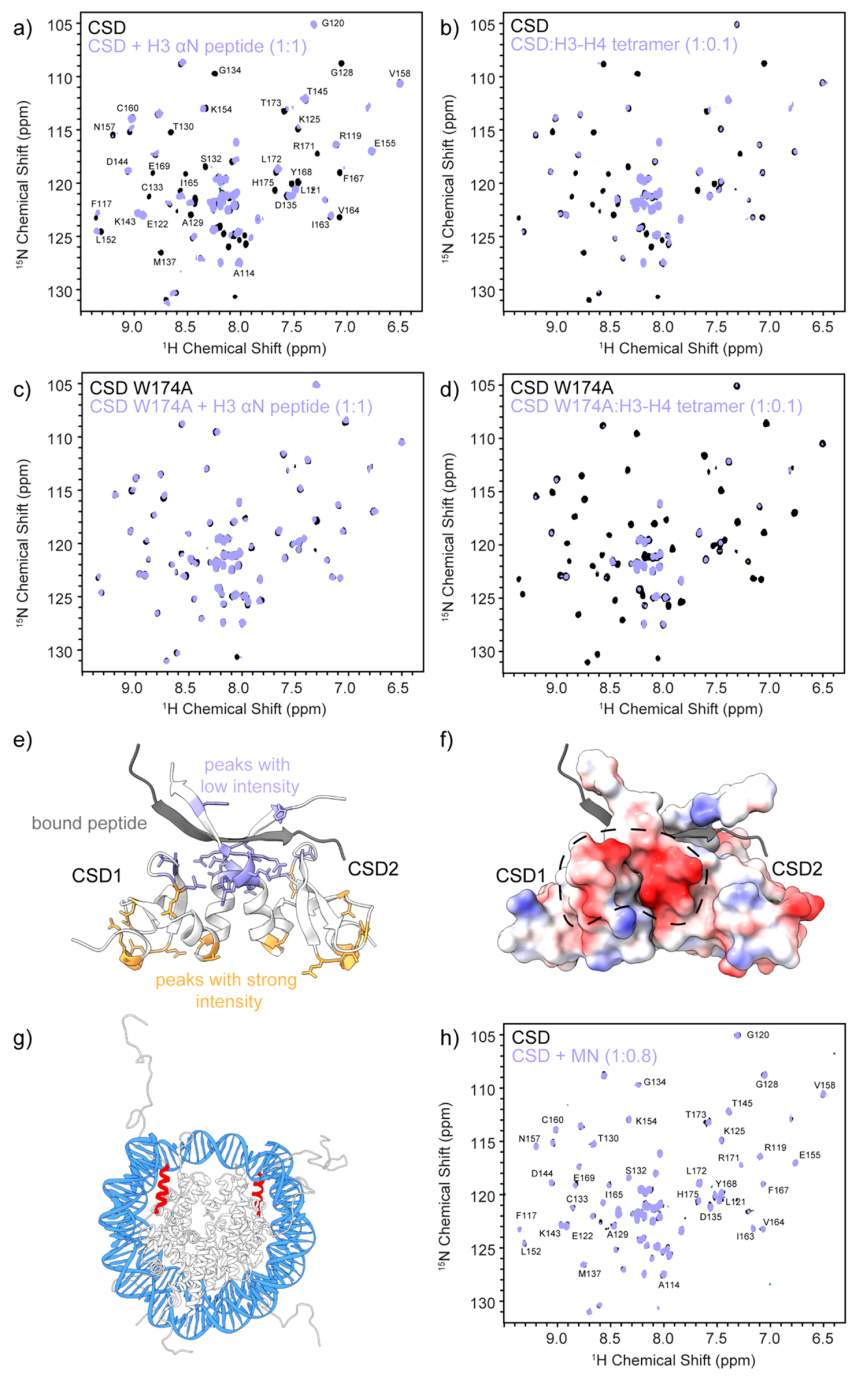
Interactions of the CSD dimer with H3 and the nucleosome. 2D HSQC experiments of a sample prepared with (a) ^15^N-labeled CSD dimer and natural abundance H3(37–59) peptide in a 1:1 ratio, (b) ^15^N-labeled CSD dimer and natural abundance H3–H4 tetramer in a 1:0.1 ratio, (c) ^15^N-labeled CSD W174A dimer and natural abundance H3(37–59) peptide in a 1:1 ratio, and (d) ^15^N-labeled CSD W174A dimer and natural abundance H3–H4 tetramer in a 1:0.1 ratio. (e) Structure of the CSD dimer with bound peptide (gray). Residues corresponding to peaks that lose intensity upon addition of the H3(37–59) peptide as determined in part a are shown in purple, while residues that retain their intensity are depicted in orange. (f) Electrostatic map of the CSD dimer illustrating a negatively charged patch that can interact with histone proteins through nonspecific interactions. (g) Structure of the nucleosome depicting the position of H3(37–59) shown in red. (h) 2D HSQC experiment of a sample containing ^15^N-labeled CSD dimer and natural abundance mononucleosomes in a 1:0.8 ratio. See Figure S7 for intensity ratio analysis.

To investigate this hypothesis, we adopted the CSD dimer point of view and performed titration HSQC experiments with the ^15^N-labeled CSD dimer and unlabeled H3 peptide or H3/H4 tetramers. The peptide encompassed residues 37–59 of H3 and included the PGTVAL motif. In both cases, we observed changes in the HSQC spectra consistent with an interaction between the CSD and the histone partner (Figure 2a,b, Figure S7a,b). In particular, we observed the disappearance of peaks at or close to the PXVXL binding surface on the dimer (Figure S8), which is also in agreement with previous binding studies.^26^ To confirm the specific nature of these interactions, we prepared a CSD dimer construct with a W174A mutation. Trp 174 forms key contacts with PXVXL residues, and its mutation to alanine has been shown to abolish binding through this motif without disrupting the assembly of the CSD dimer.^13,26^ The W174A CSD spectra did not show any changes upon addition of H3 peptide confirming the specific nature of the interaction (Figure 2c, Figure S7c). Surprisingly, however, the W174A construct was still able to bind to H3–H4 tetramers, suggesting that this interaction is not through the PXVXL-like binding motif (Figure 2d, Figure S7d). While the short H3 peptide can adopt the necessary β-strand structure upon binding to the CSD dimer (Figure 2e), the PGTVAL motif in the H3–H4 tetramer is locked into an α-helical structure and thus may not be accessible or able to rearrange into a β-sheet on the CSD dimer surface, The positively charged H3–H4 tetramer, however, may be able to interact with the CSD dimer through nonspecific electrostatic interactions. Notably, the CSD dimer has a negatively charged surface close to the PXVXL-binding interface (Figure 2f).

In the context of intact nucleosomes, nonspecific electrostatic interactions between the CSD dimer and histones would be screened by DNA (Figure 2g). In support of this hypothesis, HSQC spectra of labeled CSD dimers in the presence of intact nucleosomes showed no changes in intensity (Figure 2h, Figure S7e). No changes were also observed upon addition of tetrasomes, where DNA unwrapping may increase access to the PGTVAL motif in the αN helix of H3 (Figure S9). Therefore, it appears that the CSD dimer can bind the PGTVAL motif in H3 specifically only in the context of a short H3 peptide. In the context of full-length folded histones, the interactions appear to be nonspecific, while no binding is observed when histones are wrapped with DNA. Our results are consistent with previous literature, which indicates that the CSD dimer of pHP1β, which shares 82% sequence identity with the HP1α CSD, does not have the capacity to interact with intact nucleosomes.^24^

### pHP1α Phase Separation Slows down the Dynamics of the H3 and H4 Tails

**Figure 3:**
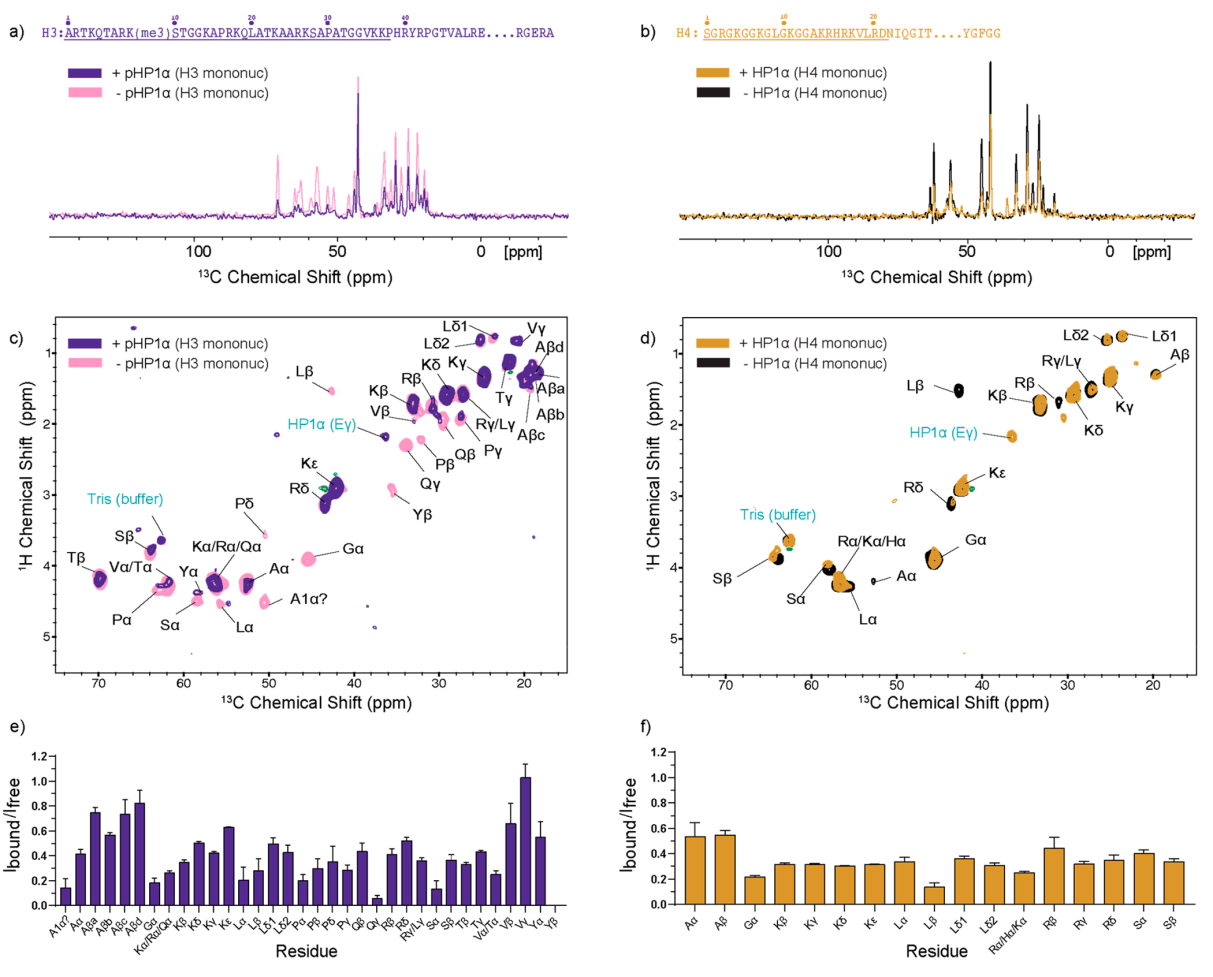
pHP1α phase separation slows the dynamics of the H3 and H4 tails. (a and b) Comparison of 1D ^1^H–^13^C INEPT MAS NMR experiments of samples containing mononucleosomes prepared with (a) ^15^N,^13^C-labeled H3 or (b) ^15^N,^13^C-labeled H4 in the presence or absence of pHP1α. (c and d) 2D ^1^H–^13^C INEPT MAS NMR experiments of the same samples. (e and f) Analysis of the peak intensity ratios of the 2D INEPT experiments in the presence (*I*_bound_) and absence (*I*_free_) of pHP1α. *I*_bound_ was corrected for the contribution of natural abundance pHP1α as described in the Experimental Section and Figure S10.

While solution NMR experiments provided a high-resolution view of the H3 tail and the CSD dimer, comprehensive studies involving full-length pHP1α and intact nucleosomes are challenging due to the large size of the species involved and the oligomerization propensity of pHP1α.^8,22^ We therefore turned to MAS NMR spectroscopy to understand the nucleosome structure and dynamics in the highly concentrated pHP1α phase separated environment. For these experiments, we prepared ^13^C,^15^N–H3 or ^13^C,^15^N–H4 labeled mononucleosomes and added a large excess of pHP1α which resulted in the formation of a cloudy condensed phase that was packed into the MAS NMR rotor (Figure S3b). We chose to work with labeled H3 and H4 as solid-state resonance assignments in the context of nucleosomes are available and previous work has shown that they yield 2D spectra with excellent resolution.^40,43^ We first recorded 1D ^1^H–^13^C INEPT experiments under magic angle spinning conditions, which allowed us to probe the dynamics of the H3 and H4 tails in phase separated pHP1α environments. In both cases, we observed a pronounced decrease in the signal for both proteins, consistent with global reduction in H3 and H4 tail dynamics (Figure 3a,b). The reduction in signal intensity is noteworthy as the phase separated sample contains a large excess of natural abundance pHP1α which has mobile regions and contributes to the INEPT intensity, especially for lysine signals (Figure S10).

To obtain a more detailed view of changes in dynamics, we extended the 1D experiments into 2D ^1^H–^13^C correlations (Figure 3c,d) and analyzed the peak intensities in the presence and absence of pHP1α phase separation (Figure 3e,f). Although some uniquely present residues can be assigned, e.g., L20, V35, and Y41 in H3 and A14 in H4, site specific assignments are not possible for most residue types in these spectra due to spectral overlap. We therefore performed the analysis by residue type. Interestingly, in H3, the ^1^H–^13^Cα correlations lose the most intensity (*I*_bound_/*I*_free_ ratio in the 0.1–0.4 range), while the side-chains remain more mobile (*I*_bound_/*I*_free_ ratio in the 0.3–1.0 range) (Figure 3e). Since ^1^H–^13^C INEPT experiments report on the Cα and side chain carbons, they can detect slightly different motions compared to ^1^H–^15^N spectra and can “see” further along the histone tail.^46^ While the V35 peaks remain relatively strong, we do observe the complete disappearance of the Y41 Cβ correlation, consistent with slower side chain motions at the base of the H3 tail in the presence of pHP1α. Compared to H3, the overall intensity reduction for H4 tail residues is more uniform, with *I*_bound_/*I*_free_ ratios in the 0.2–0.5 range for most peaks. This difference may reflect the distinct interaction modes of the histone tails with pHP1α where the methylated H3 tail interacts with the CD domain in a specific manner, while the changes in dynamics for the H4 tail may report on the much more viscous environment in the presence of phase separation. We also collected a ^1^H–^15^N INEPT spectrum for the H3 tails in the presence and absence of pHP1α (Figure S11). While not as resolved as the spectra obtained by solution NMR and presented in Figure 1b, this spectrum confirms that pHP1α interacts specifically with the H3 histone tail under these conditions.

### pHP1α Phase Separation Does Not Significantly Change the Nucleosome Core

**Figure 4:**
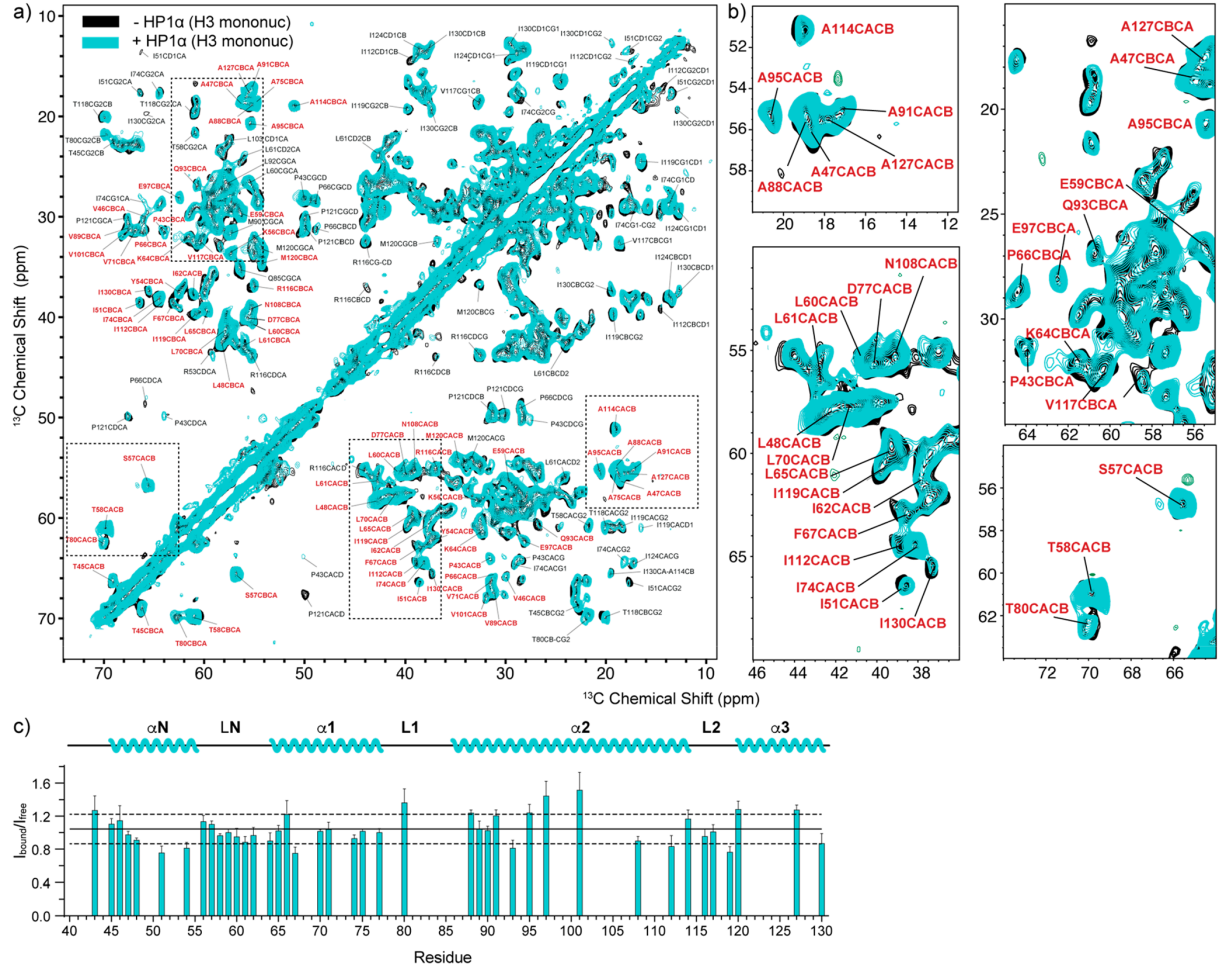
MAS NMR spectroscopy of H3 in the nucleosome core. (a) ^13^C–^13^C DARR correlations of mononucleosomes containing ^15^N,^13^C-labeled H3 in the presence (teal) and absence (black) of pHP1α phase separation. (b) Zoomed in regions of the ^13^C–^13^C DARR spectrum. (c) Analysis of the peak intensity ratios of resolved Cα–Cβ correlations in the presence (*I*_bound_) and absence (*I*_free_) of pHP1α. Only peaks labeled in red were included in the analysis. Data were collected at 750 MHz ^1^H Larmor frequency and 15 kHz MAS spinning frequency, in the presence of 20 mM Mg^2+^. The solid line represents the average of the intensity ratios, while the dashed lines represent one standard deviation above and below. Error bars were calculated as summarized in the Experimental Section.

**Figure 5:**
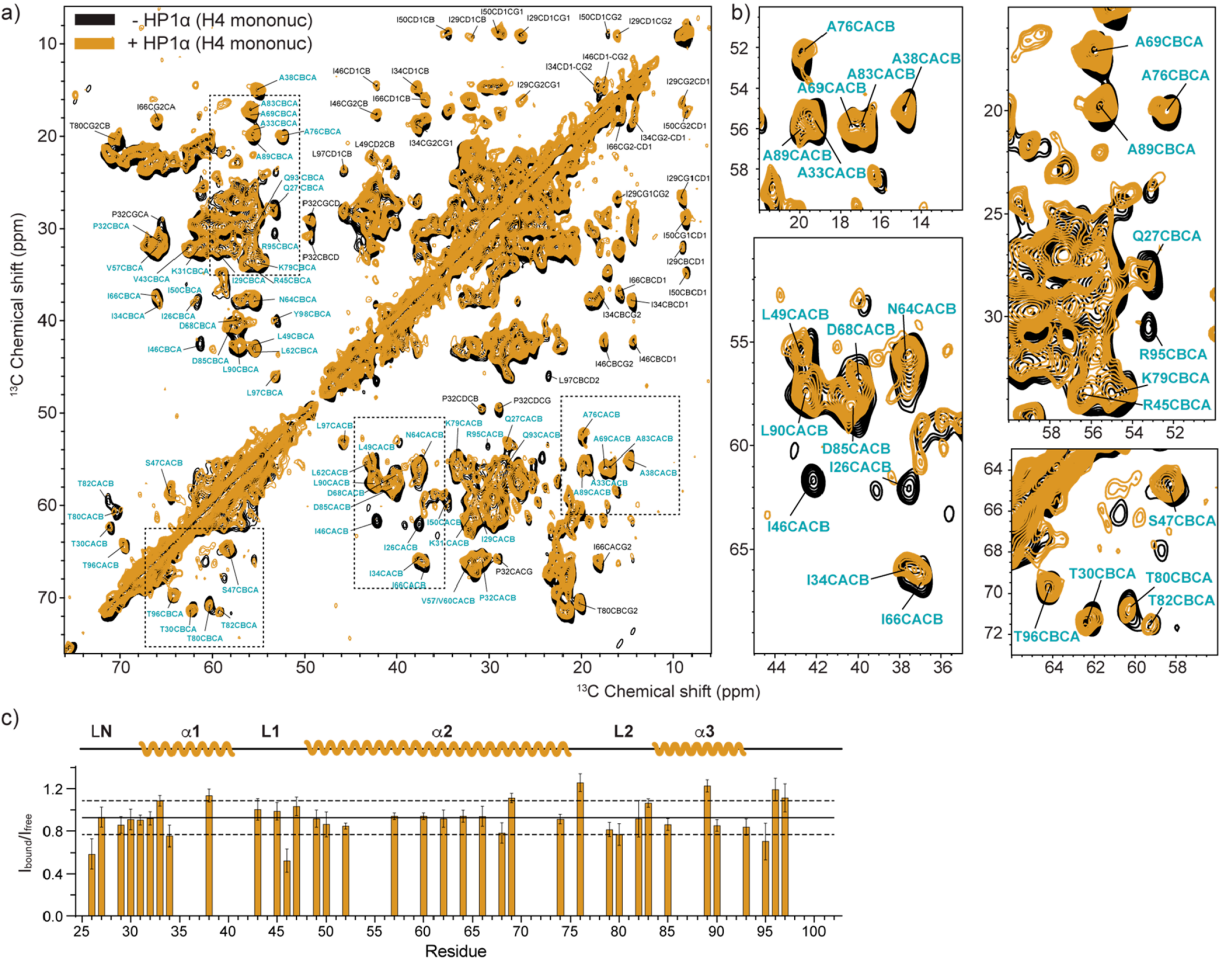
MAS NMR spectroscopy of H4 in the nucleosome core. (a) ^13^C–^13^C DARR correlations of mononucleosomes containing ^15^N,^13^C-labeled H4 in the presence (gold) and absence (black) of pHP1α phase separation. (b) Zoomed in regions of the ^13^C–^13^C DARR spectrum. (c) Analysis of the peak intensity ratios of resolved Cα–Cβ correlations in the presence (*I*_bound_) and absence (*I*_free_) of pHP1α. Only peaks labeled in blue were included in the analysis. Data were collected at 750 MHz ^1^H Larmor frequency and 11 kHz MAS spinning frequency, in the presence of 1.5 mM Mg^2+^. The solid line represents the average of the intensity ratios, while the dashed lines represent one standard deviation above and below. Error bars were calculated as summarized in the Experimental Section.

Having observed changes in the dynamics of the H3 and H4 histone tails in the presence of pHP1α, we next wondered if the phase separated environment would affect the nucleosome core. For this purpose, we recorded 2D ^13^C–^13^C DARR spectra of the H3- and H4-labeled nucleosome samples in the presence and absence of pHP1α (Figure 4 and Figure 5, respectively). DARR correlations rely on dipolar transfer between spins and are therefore well suited to characterize all carbon atoms in the relatively rigid nucleosome core.^47^ We used 20 ms of DARR mixing, which yields primarily one-bond correlations, and we took advantage of the published solid-state assignments for H3 and H4 to analyze the data.^40,43^ Qualitative comparison of the H3 and H4 nucleosome spectra in the presence and absence of pHP1α does not reveal dramatic changes in the intensity or chemical shifts of the observed peaks (Figure 4a and Figure 5a, respectively). To obtain a more quantitative picture, we compared the intensities of resolved Cα–Cβ correlations for both the H3- and H4-labeled samples (Figure 4b and Figure 5b, respectively). We used the average intensity of the symmetric Cα–Cβ and Cβ–Cα cross-peaks across the diagonal and based our analysis on 41 resolved correlations from the H3 nucleosome core (or ∼38% of the H3 core residues) and 36 resolved correlations from the H4 core (or ∼46% of the H4 core residues). While we could not include all residues in the analysis due to spectral overlap, these cross-peaks represent a substantial amount of the H3 and H4 core residues and thus provide a detailed view of any potential changes in dynamics in the nucleosome core due to the presence of pHP1α. In both cases, however, the intensity ratios for samples prepared with and without pHP1α are very similar, in the range 0.8–1.2 for most residues (Figure 4b and Figure 5b, respectively). The only intensities that significantly deviate from this trend are the Cα–Cβ correlations for Ile26 and Ile46 in H4 (ratio of 0.6 or less). Ile26 is the first H4 core residue that can be detected in H4 nucleosome spectra, while Ile46 sits in an H4 loop close to the DNA entry exit site and adjacent to Ile119 in H3. There are also some noticeable changes in the intensity for several long-range correlations in both the H3 and H4 spectra (e.g., Pro Cα–Cδ correlations in H3). However, since we did not optimize for long-range transfer in our DARR experiments, we did not include those in our analysis and focused on one-bond Cα–Cβ correlations as representatives of the overall backbone structure of the nucleosome core. Taken together, our data indicate that there are no significant changes in the overall backbone structure or dynamics of the nucleosome due to the presence of pHP1α, although several residues may experience small deviations in local motions as a result of pHP1α binding.

Finally, we note the importance of the Mg^2+^ concentration in these studies. While the solution NMR experiments presented in Figure 2 did not contain divalent cations, Mg^2+^ was required to efficiently pack the solid-state NMR samples, especially in the absence of pHP1α. Mg^2+^ has beneficial effects on dipolar spectra, as it rigidifies the nucleosome and results in improved resolution and sensitivity. At the same time, it may mask relevant changes in nucleosome dynamics upon interactions with pHP1α. Mg^2+^ may also influence the material state of the phase separated samples, as it can oligomerize nucleosomes. Fluorescence imaging of pHP1α and nucleosome samples confirmed the presence of liquid droplets even at high Mg^2+^ concentrations (Figure S12). Fluorescence recovery after photobleaching (FRAP), on the other hand, suggested that pHP1α remained dynamic at high Mg^2+^ concentrations while the nucleosomes lost their mobility but still co-localized with pHP1α into the droplets (Figure S13). This suggests that Mg^2+^-oligomerized nucleosomes may be present within pHP1α phase separated environments and may influence the availability of pHP1α-nucleosome binding sites. To account for these complications in sample preparation, spectral quality, and changes in dynamics, we recorded H3 data at 20 mM (Figure 4) and 1.5 mM (Figure S14) Mg^2+^ concentrations, as well as H4 data at 1.5 mM Mg^2+^ (Figure 5). Taken together, our data suggest that there are no substantial changes to the nucleosome core, irrespective of Mg^2+^ concentration. These conclusions also hold for phase separated pHP1α samples that contain 12-mer nucleosome arrays rather than individual mononucleosomes (Figure S15).

### Discussion

It has been suggested that HP1 proteins can exert their effects on nucleosomes through multiple mechanisms that are not mutually exclusive.^7,8,12,15−17,19,23^ First, they can directly interact with the nucleosome through their CD domain, and potentially through other segments such as the CSD domain or the hinge region.^9,10,25,26,28^ Second, HP1 proteins can compact chromatin polymers by bringing together nucleosomes through space in a manner that may directly affect DNA and histone availability.^12,14−16^ And third, they can form liquid–liquid droplets and gels that change the material properties of the surrounding nuclear environment and thus affect nucleosome dynamics indirectly.^8,12,17^ Here, we provide an atomic resolution picture of the nucleosome point of view in these complex and dynamic environments.

Our solution and solid-state NMR data collectively indicate that pHP1α primarily contacts H3K9 methylated nucleosomes through its CD domain. The interaction site comprises residues 3–11 on the methylated H3 tail, consistent with previous observations and structures.^34,35^ Notably, we do not detect any interactions between the CSD dimer interface and the PXVXL-like motif in the H3 αN helix in the context of intact nucleosomes. Our data also indicate that the nucleosome core remains intact and does not undergo substantial changes in the presence of pHP1α. Our NMR experiments focused on histones H3 and H4, and we did not directly probe interactions of pHP1α with nucleosomal DNA. However, our data present some indirect evidence that such contacts may occur. For example, in solution NMR experiments, the intensity of the V35 ^1^H–^15^N cross-peak is significantly attenuated upon titration of full-length pHP1α, while we do not observe this change in the presence of the CD domain alone. Since this residue lies at the base of the H3 tail, close to the DNA interface, it is possible that its dynamics are affected by pHP1α, and more specifically by hinge–nucleic acid interactions. In addition, we also see attenuation of several short- and medium-range ^13^C–^13^C correlations for H3 and H4 residues located at the DNA–histone interface. While more work is necessary to unveil the extent of HP1α–nucleosomal DNA interactions, we expect that in the context of phosphorylation such contacts would be secondary to interactions between the CD and the H3K9me3 modification. Indeed, previous literature has shown that phosphorylation of the NTE also severely disrupts hinge–DNA contacts.^32,33^

In its interaction patterns with the nucleosome, pHP1α appears to be much more similar to the human paralogue HP1β, rather than the yeast homologue Swi6. Previous NMR studies have shown that HP1β interacts with methylated nucleosomes only through its CD domain,^24^ while the effects of Swi6 on the nucleosome are much more profound, with remodeling of the nucleosome core and the exposure of otherwise buried residues.^27^ HP1α and HP1β share a high degree of sequence identity for the folded domains (82% for both the CD and the CSD), but they differ significantly in the NTE, hinge, and CTE (35%, 33%, and 38%, respectively).^7^ In particular, HP1β has fewer positively charged residues in the hinge, while the four phosphorylatable serine residues in pHP1α NTE are replaced by glutamic acids. Phosphorylation of HP1α, however, can bring about some similarities in the patterns of interaction of the two proteins with nucleosomes. For example, HP1β has a high preference for H3K9me3 nucleosomes over non-methylated nucleosomes,^24^ while wild-type HP1α recognizes both methylated and non-methylated nucleosomes relatively equally.^33^ HP1α NTE phosphorylation, however, increases the specificity for H3K9me3 nucleosomes approximately 6-fold by inhibiting interactions of the hinge with nucleosomal DNA and by promoting binding to the methylated H3 tail.^33^ Our data are consistent with this picture, where high specificity for H3K9me3 in mammalian paralogues is mediated by highly focused CD–H3 tail interactions and attenuated contacts to the rest of the nucleosomes surface. We note, however, that NTE phosphorylation of HP1α significantly enhances its ability to undergo liquid–liquid phase separation on its own, a property that is not shared by HP1β.^8,21^

The yeast homologue Swi6, on the other hand, displays several unique features and behaviors that distinguish it from the mammalian HP1 proteins. Its sequence identity to human HP1α is relatively low, with 11% identity for the NTE region, 42% for the CD, 10% for the hinge, and 23% for the CSD, respectively, with no substantial CTE.^7^ Notably, the Swi6 CD domain contains a loop with a sequence that mimics the H3 tail sequence, which can mediate autoinhibition and/or promote oligomerization through CD–CD interactions.^48^ In addition, the NTE and hinge regions of Swi6 are longer, presenting the opportunity for more extensive interactions with the nucleosome and thus tighter binding. Solution NMR, cross-linking, and hydrogen–deuterium exchange coupled with mass spectrometry studies suggest that Swi6 may contact nucleosomes in at least three different modalities, including CD–H3K9me3 interactions, hinge–DNA contacts, and interactions between the CSD–CSD dimer interface and a PXVXL-like motif on H2B.^27^ While Swi6 has a slight preference for H3K9me3 nucleosomes *in vitro*, phosphorylation does not appear to impart the same specificity that it does for HP1α.^33^ Collectively, these observations suggest that Swi6 has the capacity to display different modes of interactions with nucleosomes compared with pHP1α, rationalizing its much more profound effects on nucleosome structure and dynamics. These interaction pattern differences may also translate into distinct functions of Swi6 in yeast heterochromatin, which in mammalian cells may be carried out by different HP1 paralogues, PTMs, and/or other silencing proteins.

The use of solid-state NMR spectroscopy has allowed us to gain unique insights into pHP1α–nucleosome interactions under viscous, dynamic, and heterogeneous conditions of liquid–liquid phase separation. In this context, we do not see substantial changes in the nucleosome core, for either mononucleosomes or nucleosome arrays. Instead, we observe a pronounced and global decrease in INEPT signals for both the H3 and H4 tails. While the CD domain can slow down the motion of the H3 tail through binding interactions, the decrease in H4 tail dynamics is particularly noteworthy, as the H4 tail is not known to interact specifically with HP1 proteins. The decrease in motion could be due to nonspecific interactions with the abundant pHP1α proteins surrounding the nucleosome, or it could be a reflection of modified DNA–tail contacts in the presence of pHP1α. Nevertheless, such a global decrease in tail dynamics may have important consequences for histone PTM readers, writers, and erasers that need access to these mobile and disordered histone segments for binding and regulation.^5^

pHP1α forms condensates through phosphorylated NTE–hinge contacts, which can build a large network of electrostatic interactions between pHP1α dimers.^8,13^ Previous work has shown that HP1α and pHP1α can phase separate with H3K9me3 nucleosome arrays to a similar extent and that addition of arrays can significantly reduce the saturation concentration necessary to observe LLPS.^21^ Furthermore, the addition of nucleosome polymers promotes a more dynamic environment and slows down gelation of pHP1α droplets.^22^ In our solid-state NMR samples, we have 20–30-fold excess of pHP1α dimers compared to methylated H3 tails, and we, therefore, expect that the majority of pHP1α proteins interact with other pHP1α molecules rather than nucleosome tails. The availability of other pHP1α dimers in the vicinity may sequester hinge regions from the nucleosome, further increasing the specificity of CD–H3K9me3 interactions. Interestingly, higher order oligomerization also appears to increase the specificity of Swi6 toward methylated nucleosomes in live *S. pombe* cells.^49^

## Conclusion

In summary, our NMR structural data suggest that the interactions of pHP1α with H3K9me3 nucleosomes are highly specific, with the CD domain serving as the primary contact to methylated histone H3 tails under dilute conditions. In phase separated environments, the presence of pHP1α leads to a global reduction in motion for the histone tails but does not cause observable changes in the structure of the nucleosome core. This behavior is in stark contrast to the yeast homologue Swi6 but similar to the interaction patterns of human pHP1β with methylated nucleosomes.^24,27^ Considering that pHP1α is constitutively expressed in cells,^33^ our study raises important questions regarding the functional consequences of phosphorylation in cells and how pHP1α might balance nonspecific electrostatic interactions that lead to LLPS with highly specific contacts that mediate chromatin transactions.

## Supporting Information Available

Additional experimental details, materials, and methods, including analysis of the prepared materials, microscopy images of the samples, binding assays, and additional solid-state and solution NMR data and analysis (PDF)
