# Supplementary material for: Phosphorylated HP1α–Nucleosome Interactions in Phase Separated Environments
Source: J Am Chem Soc. 2023 Oct 23;145(44):23994–4004. doi: 10.1021/jacs.3c06481 (PMC10636758; doi:10.1021/jacs.3c06481)
Supplement: Supplementary file 1 — ja3c06481_si_001.pdf [file ja3c06481_si_001.pdf]

Supporting Information

Phosphorylated HP1α-nucleosome interactions in phase separated environments

Nesreen Elathram, Bryce E. Ackermann, Evan T. Clark, Shelby R. Dunn, Galia T. Debelouchina*

Affiliation for all authors:
Department of Chemistry and Biochemistry
University of California, San Diego
La Jolla, CA 92093
U.S.A

** Corresponding author: gdebelouchina@ucsd.edu*

Experimental Section

Lab Techniques

All commonly used reagents were purchased from Sigma-Aldrich or Fisher Scientific. Isotopically enriched reagents were purchased from Cambridge Isotope Laboratories. Dialysis kits were purchased from Thermo Fisher Scientific and protein concentrators were obtained from Sartorius. Reverse-phase (RP) HPLC was performed on a 2545 Binary Gradient Module Waters system equipped with a 2484 UV/vis detector. For prep-scale RP-HPLC purification, we relied on a Waters XBridge BEH C18 19 mm x 250 mm, 10 µm particle size column, while analytical measurements were performed using a Symmetry300 C18 4.6 mm x 150 mm, 5 µm particle size column. HPLC solvent A contained 100% H_2O + 0.1% trifluoroacetic acid (TFA), while solvent B contained 100% acetonitrile + 0.1 % TFA. For size-exclusion chromatography, a Superdex 200 10/300 column was used with an ÄKTA pure protein purification system (GE Healthcare Life Sciences/Cytiva). For ion-exchange, we used a 5 mL HiTrap SP HP column on the ÄKTA pure protein purification system (GE Healthcare Life Sciences/Cytiva). LC-ESI-TOF MS analysis was conducted on an Agilent 6230 Accurate-Mass TOFMS. Gel images were acquired using a camera and light box from Fotodyne Incorporated. Protein absorbances were measured using a Nanodrop One Spectrophotometer by Thermo Scientific.

Constructs

For mononucleosome assembly, we used the following 177 bp DNA sequence (with the 147 bp strong nucleosome positioning "601" sequence (1) highlighted in bold):

ACTACGCGGCCGCC**CTGGAGAATCCCGGTGCCGAGGCCGCTCAATTGGTCGTAGACAGCTCTAG
CACCGCTTAAACGCACGTACGCGCTGTCCCCCGCGTTTTAACCGCCAAGGGGATTACTCCCTAG
TCTCCAGGCACGTGTCAGATATATACATCCTGT**GCATGTAAGATCCAGT

The HP1α chromodomain and chromoshadow domain genes were excised from a GST HP1α plasmid provided by Naoko Tanese (2) (Addgene plasmid #24074; http://n2t.net/addgene:24074; RRID:Addgene_24074), and cloned into a pET vector backbone of a 2BT MacroLab plasmid (generously provided by Dr. Kevin Corbett) using NEBuilder® HiFi DNA Assembly Master Mix. The resulting plasmids contained the following His6-TEV-CD or His6-TEV-CSD-CTE constructs:

CD construct:
MKSSHHHHHHENLYFQ SEEYVVEKVL DRRVVKGQVE YLLKWKGFSE EHNTWEPEKN
LDCPELISEF MKKYKKMKE

CSD-CTE construct:
MKSSHHHHHHENLYFQ SNDIARGFER GLEPEKIIGA TDSCGDLMFL MKWKDTDEAD
LVLAKEANVK CPQIVIAFYE ERLTWHAYPE DAENKEKETA KS

Preparation of histone proteins and DNA

Isotopically labeled histone H3 K9C was expressed and purified according to the protocol described in ref. (3) with some modifications. Briefly, a plasmid bearing the histone H3.1

K9C, C96A, C110A gene was transformed in BL21(DE3)-Rosetta cells and pre-cultures were grown at 37 °C overnight under kanamycin antibiotic selection in LB media. Pre-cultures were pelleted, LB was removed, and the pellet was resuspended in ^{13}C,^{15}N-enriched M9 media, then added to 1L of ^{13}C,^{15}N-enriched M9 expression media. The cells were grown at 37 °C until OD600 = 0.6 and induced with 0.5 mM IPTG. Cells were collected 3 hours later by centrifugation at 5,000 xg for 20 minutes. The cell pellet was lysed, and the inclusion body was washed four times with lysis buffer (20 mM Tris pH = 7.6 at 4 °C, 200 mM NaCl, 1 mM EDTA, 1 mM 2-mercaptoethanol) + 0.1% v/v of Triton-X. The inclusion body pellet was resuspended in suspension buffer (6 M guanidine, 20 mM Tris, 1 mM EDTA, 100 mM NaCl, 1 mM DTT, pH 7.5 at 4 °C), incubated at 4 °C for 120 min, and centrifuged for 30 min at 27,000 xg. The supernatant was dialyzed against urea dialysis buffer (7 M urea, 10 mM Tris, 1 mM EDTA, 100 mM NaCl, 1 mM DTT, pH 7.5 at 4 °C), and purified with ion exchange chromatography. The fractions containing H3 K9C protein were treated with 10 mM TCEP for 30 min, the pH was adjusted to ~ 2, and the sample was loaded onto a prep-size column and subjected to RP-HPLC. Pure fractions were collected using a 30%-70% solvent B gradient. Isotopically labeled H4 was prepared in a similar manner omitting the incubation step with TCEP before RP-HPLC purification.

Natural abundance human histones were expressed in BL21(DE3)-Rosetta cells. Cells were grown at 37 °C until OD600 = 0.6 and protein expression was induced with 0.5 mM IPTG. Cells were collected 3 hours later by centrifugation at 5,000 xg. Purification was performed as described above. All pure histone preparations were lyophilized and stored at –20 °C. Protein purity was assessed by SDS-PAGE, analytical RP-HPLC and ESI-MS. A plasmid containing 12 repeats of the 601 DNA sequence separated by 30 bp linkers (12 x 601) was prepared in mg quantities according to the protocol described in Ref.(3). This construct contains EcoRV restriction enzyme sites to produce the 12x601 DNA construct for array reconstitution and ScaI restriction enzyme sites to produce a 177 bp 601 DNA construct for mononucleosome assembly. After digestion with the appropriate enzyme (New England Biolabs), the relevant construct was purified with 7% PEG6000 and ethanol precipitation. The DNA pellets were re-dissolved in 10 mM Tris, pH 8.0, 0.1 mM EDTA buffer and stored at –20 °C.

Histone H3 K9C trimethylation

H3 K9C trimethylation was performed according to ref. (4). 25 mg of H3 K9C were reduced in alkylation buffer (1 M HEPES, 4 M guanidium-HCl, 10 mM D/L-methionine, 20 mM DTT, pH 7.8) for 1 hr at 37 °C. To perform the alkylation reaction, the sample was incubated with 400 mM (2-bromoethyl)-trimethylammonium bromide (Sigma) in the dark for 2.5 hrs with mixing. Then, fresh DTT was added and the reaction was left in the dark for another 2.5 hrs. After verifying reaction completion by analytical RP-HPLC, the reaction was quenched with 700 mM 2-mercaptoethanol. The product was purified by prep scale RP-HPLC and confirmed by ESI-TOFMS.

Histone octamer and tetramer assembly

Histone octamers were assembled according to ref.(3). Briefly, lyophilized histones were dissolved by the addition of unfolding buffer (6 M guanidinium HCl, 20 mM Tris, 5 mM

DTT, pH 7.6 at 4 °C). The histones were combined in a 1.1:1.1:1:1 (H2B:H2A:H4:H3) molar ratio and were dialyzed against refolding buffer (2 M NaCl, 10 mM Tris, 1 mM EDTA, 1 mM DTT, pH 7.6 at 4 °C). The assembled octamers were purified by size-exclusion chromatography and stored in 50% glycerol at -20 °C. The octamer purity was assessed on an 18% SDS-PAGE gel. Histone tetramers were prepared analogously except that H2A and H2B were not used.

Mononucleosome assembly and 12x601 array assembly
Mononucleosomes were reconstituted by combining 177 bp 601 DNA and histone octamers to a total volume of ~20 ml of 2.5 µM 601 DNA in 2M TEK buffer (2 M KCl, 10 mM Tris pH 7.5 at 4 °C, 0.1 mM EDTA, 1 mM DTT), followed by salt gradient dialysis against 10 mM TEK buffer (10 mM KCl, 10 mM Tris pH 7.5 at 4 °C, 0.1 mM EDTA, 1 mM DTT), for ~24 hr at 4 °C. The appropriate ratio of DNA to octamer was optimized on a smaller scale so that well-formed nucleosomes without excess free DNA could be obtained. The mononucleosome assembly was visualized on native 5% TBE or APAGE gel (2% acrylamide/1% agarose) stained with SybrGold (Life Technologies).

12-mer nucleosome arrays were prepared in a similar manner except that 0.3 eq of the 155 bp MMTV buffer DNA were added to the 12x601 DNA/octamer mix. This buffer DNA ensures that all twelve 601 sites are occupied by nucleosomes while excess octamers are deposited on the MMTV DNA. After dialysis, the MMTV mononucleosomes were purified by precipitation with 2 mM Mg^{2+}. Samples were visualized on native APAGE gels (2% acrylamide/1% agarose) stained with SybrGold.

Tetrasomes were prepared analogously except that DNA was mixed with H3-H4 tetramers in a 1.8:1 DNA to tetramer ratio. The presence of tetramers was confirmed by reference to the gel retention behavior described in Ref. (5). These samples were kept at low salt (10 mM KCl) during all experiments.

Expression and purification of full-length HP1α and related constructs
Full-length phosphorylated HP1α was expressed and purified according to ref. (6). Plasmids encoding for HP1α and the CK2 kinase were co-transformed in BL21(DE3)-Rosetta cells. The cells were grown at 37 °C under ampicillin (100 μg/mL) and streptomycin (50 μg/mL) selection in LB until OD600=0.6. At this point, the culture flasks were transferred to 18 °C and protein expression was induced with 0.5 mM IPTG for 20 hours. Cells were collected by centrifugation at 5,000 xg for 25 minutes. The cell pellet was resuspended in lysis buffer (20 mM HEPES pH 7.2, 300 mM KCl, 10% glycerol, EDTA-free protease inhibitor tablet) and sonicated at 4 °C. The cell lysate was centrifuged for 20 minutes at 30,000 xg and the supernatant was mixed with Ni-NTA resin (1 ml of beads for 20 mg of protein, Thermo Scientific) for 1 hour at 4 °C. The mixture was loaded onto a Bio-Rad Econo-column and washed with wash buffer (20 mM HEPES pH 7.2, 300 mM KCl, 10% glycerol, 40 mM imidazole) and then eluted with elution buffer (20 mM HEPES pH 7.2, 300 mM KCl, 400 mM imidazole). The elution was incubated with TEV-protease during dialysis against imidazole-free buffer, then purified by RP-HPLC followed by lyophilization. To refold the protein, pure lyophilized HP1α was added to 6 M guanidinium HCl, 20 mM HEPES pH 7.2, 75 mM KCl, 1 mM DTT, and the solution was

dialyzed in 1 M guanidinium HCl, 20 mM HEPES pH 7.2, 150 mM KCl and 1 mM DTT for 4 – 6 hr. Final dialysis was performed against 0 M guanidinium HCl, and the protein was concentrated to 1-2 mM. The chromodomain (CD) and chromoshadow domain (CSD-CTE) constructs were expressed and purified following the same protocol except that co-expression with the CK2 kinase was not necessary.

Sample preparation for NMR spectroscopy
For solid-state NMR experiments, freshly prepared mononucleosome samples (~20 ml, 2.5 μM) were concentrated to a final volume of 5 mL in 10 mM Tris, 10 mM KCl, 0.1 mM EDTA, 1 mM DTT, pH 7.5 at 4 °C. 15-20 mg of freshly refolded phosphorylated HP1α (2 mM stock solution), and 0.01% NaN_3 were added to the mononucleosome samples. The Mg^{2+} concentration was adjusted to 20 mM (H3 mononucleosome samples), 1.5 mM (H4 mononcuelsome samples), and 6 mM (H4 array samples). The sample was mixed and incubated for 10 mins on ice before centrifugation at 70,000 rpm (~500,000 xg) for 23 hr at 4 °C using an NVT 90 Beckman ultracentrifuge rotor. The nucleosome pellet was then transferred to the NMR rotor using a plunger pipette. The final amount of sample packed into the NMR rotor was determined by measuring the A260 absorbance of the leftover supernatant and subtracting it from the A260 absorbance before centrifugation. The A260 absorbance reports on the amount of mononucleosomes that are present in the sample/supernatant. Nucleosome arrays were prepared in a similar manner.

For the solution NMR experiments presented in Fig. 1 and Fig. S6 (mononucleosome titrations with full-length HP1α and CD domain), samples were prepared in the following way. Freshly prepared mononucleosome samples (~10 mL, 2.5 µM) were concentrated to a final volume of 150 µL in 10 mM Tris, 10 mM KCl, 0.1 mM EDTA, 1 mM DTT, pH 7.5 at 4 °C, and transferred to a 3 mm NMR tube. 10% D_2O and 0.01% NaN_3 were added to the tube and a control ^{1}H-^{15}N HSQC experiment was obtained. For each subsequent titration experiment, an appropriate volume of refolded phosphorylated HP1α (1 mM stock solution) was added to the tube so that the final HP1α concentration was 50 µM, 100 µM or 150 µM. CD domain titration samples were prepared in the same manner except that the final concentration of the CD domain was 150 µM or 500 µM. For the solution NMR experiments presented in Fig. 2, Fig. S7 and Fig. S9 (CSD dimer experiments with peptides, tetramers, mononucleosomes, and tetrasomes), samples were prepared in 20 mM HEPES pH 7.2, 75mM KCl, 1 mM DTT with 10% D_2O.

Solution NMR spectroscopy
Solution NMR spectroscopy experiments were performed on a Bruker Avance Neo 800 MHz NMR spectrometer equipped with a triple resonance TXO cryoprobe. The ^{1}H-^{15}N HSQC spectra were collected with a 150 µM ^{13}C,^{15}N-H3 K9Cme3 labeled mononucleosome sample with varying amounts of phosphorylated HP1α or CD domain at natural abundance. Experiments were performed at 298 K with the standard Bruker fhsqcf3gpph pulse sequence. The experiments were collected with 94 ms acquisition in the direct dimension, 128 points with 22 ms acquisition in the indirect dimension, 416 scans for all experiments except for the experiment containing 150 µM of full-length HP1α, which was obtained with 320 scans. For quantitative analysis of the titration experiments, peak intensities were normalized based on the number of scans when

applicable. Other experimental parameters include J_{NH} = 92 Hz, ^{1}H transmitter frequency offset = 4.7 ppm, ^{15}N transmitter frequency offset = 120 ppm, and interscan delay of 1.5 s.

For CSD binding studies, ^{1}H-^{15}N TROSY-HSQC spectra were acquired using the standard Bruker trosyf3gpph19 pulse sequence. Experiments used the following parameters: interscan delay of 1.25 s, ^{1}H center frequency of 4.7 ppm, ^{15}N center frequency of 120 ppm, and J_{NH} = 90 Hz. Further experimental details are presented in **Table S1** below.

Solid-state NMR spectroscopy
The solid-state NMR experiments were conducted on a 750 MHz NMR spectrometer, equipped with an AVANCE II Bruker console and a triple resonance (^{1}H, ^{13}C, ^{15}N) 3.2 mm MAS Bruker probe (PH MAS DVT 750SB EFREE BL3.2). All experiments were performed using 3.2 mm thin-wall Bruker rotors with an MAS spinning frequency of 15 kHz (H3 mononucleosome experiments) or 11.1 kHz (H4 mononucleosome or array experiments). KBr was used to set the magic angle and adamantane (40.49 ppm) was used as a ^{13}C chemical shift reference (7). The temperature of the cooling nitrogen gas was set to 275 K (DARR experiments) or 295 K (INEPT experiments) while we estimate that the actual sample temperature during the MAS experiments is 10 – 15 K higher. The experimental details for each sample are summarized in **Tables S2-S3** below.

NMR Data Analysis
NMR spectral data were processed with TopSpin 4.0.5 and analyzed using NMRFAM-SPARKY and CCPNMR 3.0.2 (8, 9). Nucleosome images were rendered using the Chimera software (10). Solid-state resonance assignments for the DARR spectra of histone H3 and histone H4 were transferred from ref. (11, 12). The ^{1}H-^{15}N HSQC cross-peaks of the H3 tail were assigned according to ref. (13). The quantitative analysis of Cα-Cβ peak intensities was performed by extracting the peak height for each cross-peak on both sides of the diagonal and taking the average. Only resolved unambiguous cross-peaks were included in the analysis. Error bars were calculated by extracting the signal-to-noise data for each cross-peak and using the following equation to estimate the relative uncertainty in the peak height ratio:

$$relative\ uncertainty = \sqrt{(\frac{\sigma_{bound}}{I_{bound}})^2 + (\frac{\sigma_{free}}{I_{free}})^2}$$

Here, I_{bound} and I_{free} represent the intensities of the cross-peaks in the pHP1α-bound and pHP1α-free spectra, respectively. σ_{bound} and σ_{free} are the uncertainties in peak heights in the bound and free states, respectively.

The intensities ratios presented in Fig. 3 were analyzed in the following way. First, we ensured that each sample had the same amount of mononucleosomes and was recorded using the same experimental conditions (see Table S3). For each set of experiments, H3-based or H4-based, we measured the intensity of each peak in the control sample (I_{free}) and in the sample containing pHP1α (I_{bound}'). The spectra of samples containing pHP1α display a unique peak Eγ, which results from natural abundance signals from the

glutamate residues present in pHP1α's disordered regions. As the H3 and the H4 tails do not contain glutamate residues, this peak can, therefore, be used to estimate and correct for the natural abundance contribution of pHP1α to I_{bound}'. To do so, we recorded a spectrum of phase separated pHP1α without mononucleosomes (grey in **Fig. S10**) and scaled the intensity of the spectrum, so that the intensities of the Eγ peaks in the spectra with or without mononucleosomes were equal. After scaling, significant intensity remained, e.g. for lysine correlations. That intensity was subtracted from I_{bound}' to give the corrected value I_{bound}, which was used to calculate the intensity ratios presented in Fig. 3e,f.

Fluorescence imaging experiments of LLPS samples

Fluorescence microscopy and fluorescence recovery after photobleaching (FRAP) experiments were conducted using a Leica SP8 confocal microscope equipped with a 40X oil objective. To prepare LLPS droplets, pHP1α (100 μM or 150 μM depending on the experiment, see figure captions) was mixed with mononucleosomes (3.3 μM, 5 μM or 100 μM depending on the experiment, see figure captions) in buffer consisting of 10 mM Tris, 0.1 mM EDTA, 10 mM KCl, pH 7.5. The sample was thoroughly mixed, and 9 μL were placed on a microscope glass slide. Cy3-labeled pHP1α (5%) and DNA-binding dye (YOYO-1) (1 μM) were used for fluorescence. These were excited by 552 nm and 488 nm lasers, respectively. For photobleaching, a circular region of 3-4 μm was manually selected and was subjected to 100% laser power at its center, then monitored for fluorescence recovery over 1.5 minutes. The acquired raw data underwent correction for photobleaching during acquisition and were normalized to the initial signal. The error bars represent the standard deviation based on data from 10 or more measurements. The recovery curves were fitted to a two-phase decay model using GraphPad Prism version 9.0. Image analysis was carried out using FIJI.

References

1. P. T. Lowary, J. Widom, New DNA sequence rules for high affinity binding to histone octamer and sequence-directed nucleosome positioning. *J Mol Biol* **276**, 19-42 (1998).
2. M. F. Vassallo, N. Tanese, Isoform-specific interaction of HP1 with human TAFII130. *Proc Natl Acad Sci U S A* **99**, 5919-5924 (2002).
3. P. N. Dyer *et al.*, Reconstitution of nucleosome core particles from recombinant histones and DNA. *Methods Enzymol* **375**, 23-44 (2004).
4. M. D. Simon *et al.*, The Site-Specific Installation of Methyl-Lysine Analogs into Recombinant Histones. *Cell* **128**, 1003-1012 (2007).
5. E. A. Morrison, L. Baweja, M. G. Poirier, J. Wereszczynski, C. A. Musselman, Nucleosome composition regulates the histone H3 tail conformational ensemble and accessibility. *Nucleic Acids Research* **49**, 4750-4767 (2021).
6. B. E. Ackermann, G. T. Debelouchina, Heterochromatin Protein HP1α Gelation Dynamics Revealed by Solid-State NMR Spectroscopy. *Angew. Chem. Int. Ed.* **58**, 6300-6305 (2019).
7. C. R. Morcombe, K. W. Zilm, Chemical shift referencing in MAS solid state NMR. *J Magn Reson* **162**, 479-486 (2003).
8. S. P. Skinner *et al.*, CcpNmr AnalysisAssign: a flexible platform for integrated NMR analysis. *J Biomol NMR* **66**, 111-124 (2016).

9. W. Lee, M. Tonelli, J. L. Markley, NMRFAM-SPARKY: enhanced software for biomolecular NMR spectroscopy. *Bioinformatics* **31**, 1325-1327 (2015).
10. E. F. Pettersen *et al.*, UCSF Chimera--a visualization system for exploratory research and analysis. *J Comput Chem* **25**, 1605-1612 (2004).
11. X. Shi, C. Prasanna, K. Pervushin, L. Nordenskiöld, Solid-state NMR 13C, 15N assignments of human histone H3 in the nucleosome core particle. *Biomolecular NMR Assignments* 10.1007/s12104-020-09927-w (2020).
12. X. Shi *et al.*, Structure and Dynamics in the Nucleosome Revealed by Solid-State NMR. *Angew Chem Int Ed Engl* **57**, 9734-9738 (2018).
13. E. A. Morrison, S. Bowerman, K. L. Sylvers, J. Wereszczynski, C. A. Musselman, The conformation of the histone H3 tail inhibits association of the BPTF PHD finger with the nucleosome. *Elife* **7**, e31481 (2018).
14. J. Kang *et al.*, Mitotic centromeric targeting of HP1 and its binding to Sgo1 are dispensable for sister-chromatid cohesion in human cells. *Mol Biol Cell* **22**, 1181-1190 (2011).
15. X. Shi, C. Prasanna, K. Pervushin, L. Nordenskiold, Solid-state NMR (13)C, (15)N assignments of human histone H3 in the nucleosome core particle. *Biomol NMR Assign* 10.1007/s12104-020-09927-w (2020).

Supporting Figures

a) **HP1α domain organization**

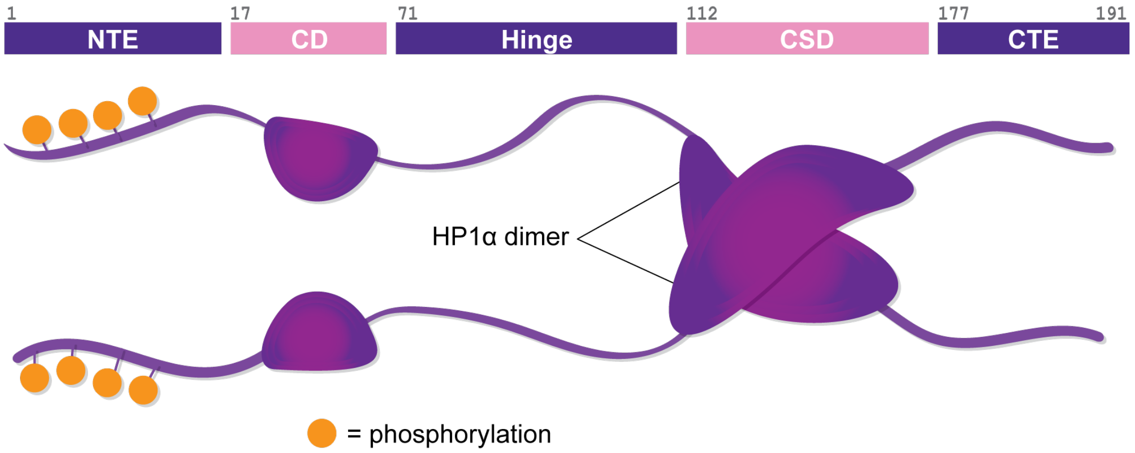

SGKKTKRTAD SSSSEDEEEY VVEKVLDRRV VKGQVEYLLK WKGFSEEHNT WEPEKNLDCP ELISEFMKKY
KKMKEGENNK PREKSESNKR KSNFSNSADD IKSKKKREQS NDIARGFERG LEPEKIIGAT DSCGDLMFLM
KWKDTDEADL VLAKEANVKC PQIVIAFYEE RLTWHAYPED AENKEKETAK S

b) **H3 Sequence**

ARTKQTARK(me3)S TGGKAPRKQL ATKAARKSAP ATGGVKKPHR YRPGTVALRE IRRYQKSTEL LIRKLP-
FQRL VREIAQDFKT DLRFQSSAVM ALQEAAEAYL VGLFEDTNLA AIHAKRVTIM PKDIQLARRI RGERA

= H3 tail = H3 core

c) **H4 Sequence**

SGRGKGGKGL GKGGAKRHRC VLRDNIQGIT KPAIRRLARR GGVKRISGLI YEETRGVLKV FLENVIRDAV
TYTEHAKRKT VTAMDVVYAL KRQGRTLYGF GG

= H4 tail = H4 core

Figure S1. a) Domain architecture and sequence of the HP1α dimer. NTE – N-terminal extension, CD – chromodomain, CSD – chromoshadow domain, CTE – C-terminal extension. b) Sequence of histone H3.1 used in this study. The position of the dynamic H3 tail is shown in light blue. The native cysteine residues at positions 96 and 110 were mutated to alanine, so that cysteine alkylation at position 9 could be performed. c) Sequence of histone H4. The position of the dynamic H4 tail is shown in light green.

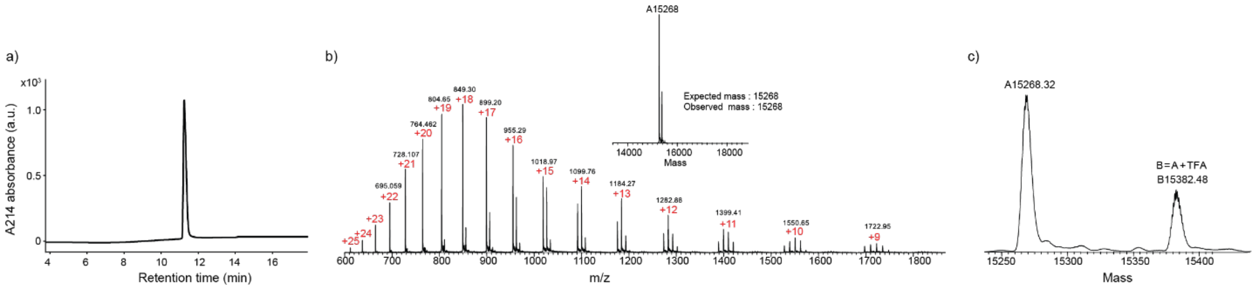

Figure S2. a) Representative RP-HPLC chromatogram of purified natural abundance H3 K9me3 prepared as a cysteine methylation mimic. b) ESI-MS analysis of the purified protein. c) Deconvoluted mass spectrum, showing a peak with the expected mass and a TFA adduct.

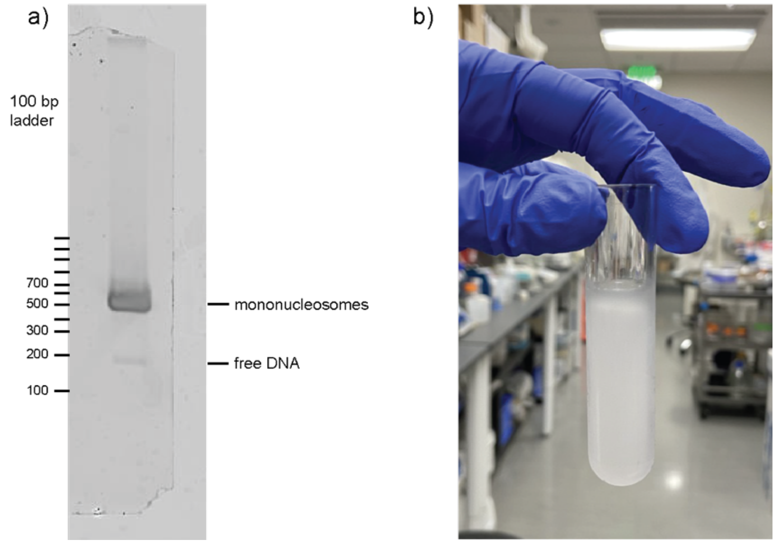

Figure S3. a) Native 5% TBE gel of a representative mononucleosome sample. b) Phase separation of a representative sample containing mononucleosomes and pHP1α before packing into the NMR rotor for MAS experiments.

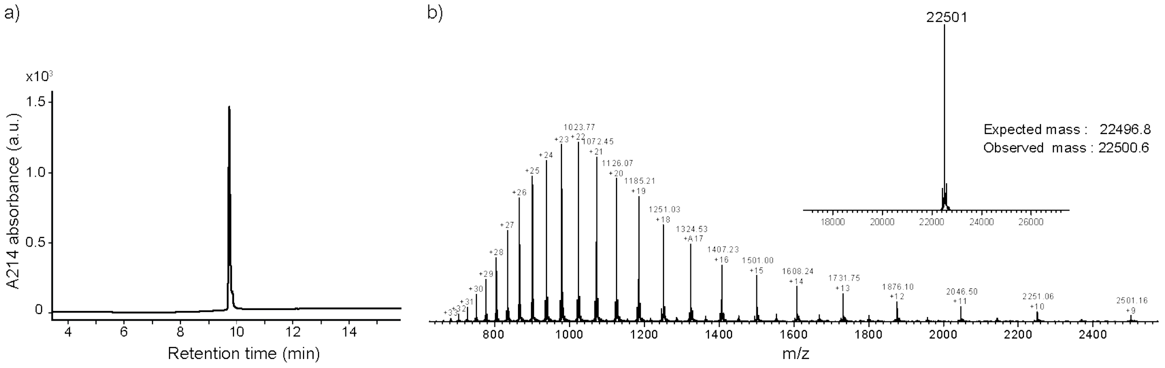

Figure S4. a) Representative RP-HPLC chromatogram of purified pHP1α. b) ESI-MS analysis of the purified protein. The expected mass corresponds to HP1α with four phosphorylated serine residues.

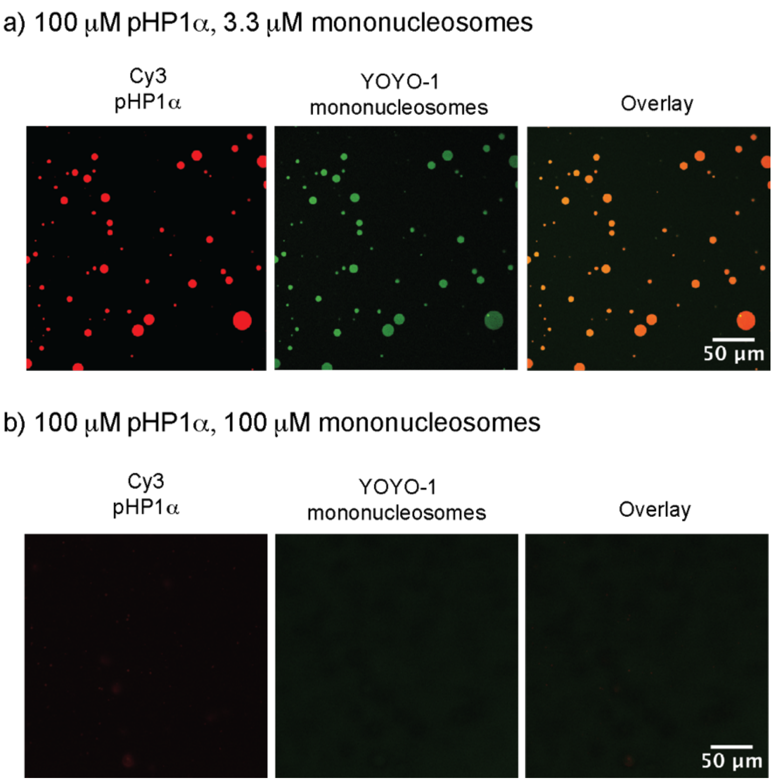

Figure S5. a) Control sample demonstrating phase separation and co-localization of pHP1α with nucleosomes at a ratio of 30:1. b) Samples prepared at a 1:1 ratio of pHP1α to nucleosomes do not show phase separation. This ratio matches the highest ratio used in our solution NMR experiments presented in Figure 1b. All experiments were performed in 10 mM Tris, 0.1 mM EDTA, 10 mM KCl, pH 7.5 to match the solution NMR conditions. Samples were visualized with 5% Cy3-labeled pHP1α and 1 μM YOYO-1 dye, which reports on the location of DNA.

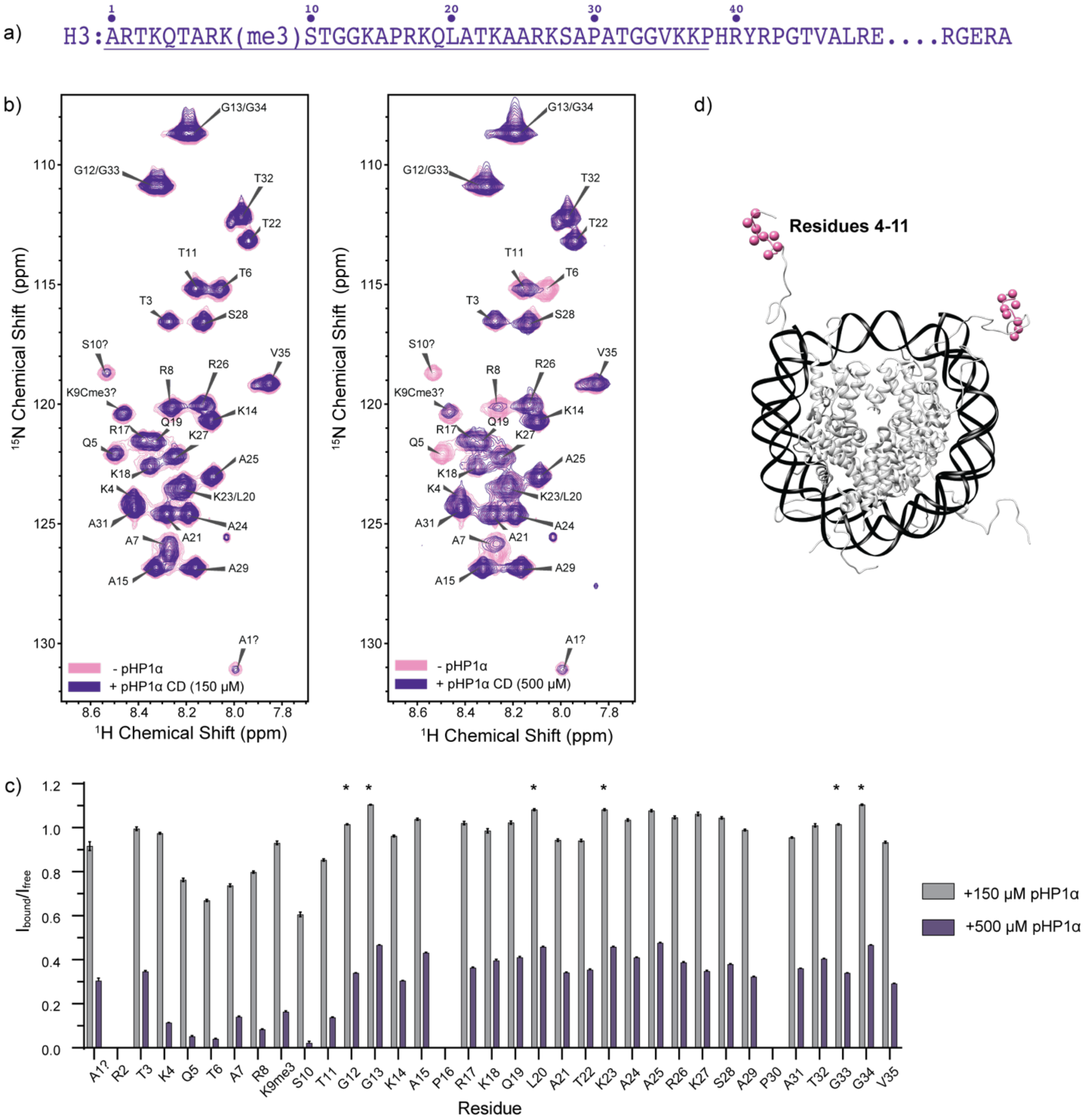

Figure S6. a) H3 sequence where the underlined residues mark the dynamic N-terminal tail. b) ^{1}H-^{15}N HSQC experiments of 150 μM ^{13}C,^{15}N-H3 labeled mononucleosomes with two different concentrations of the CD domain of HP1α. c) Analysis of the peak intensities in the spectra shown in b). The asterisks denote overlapped peaks where analysis for the individual residue could not be performed, i.e. G13/G34, G12/G33, L20/K23. Error bars are calculated based on the signal-to-noise for each cross-peak. d) H3 tail residues that experience significant changes in intensity upon CD binding.

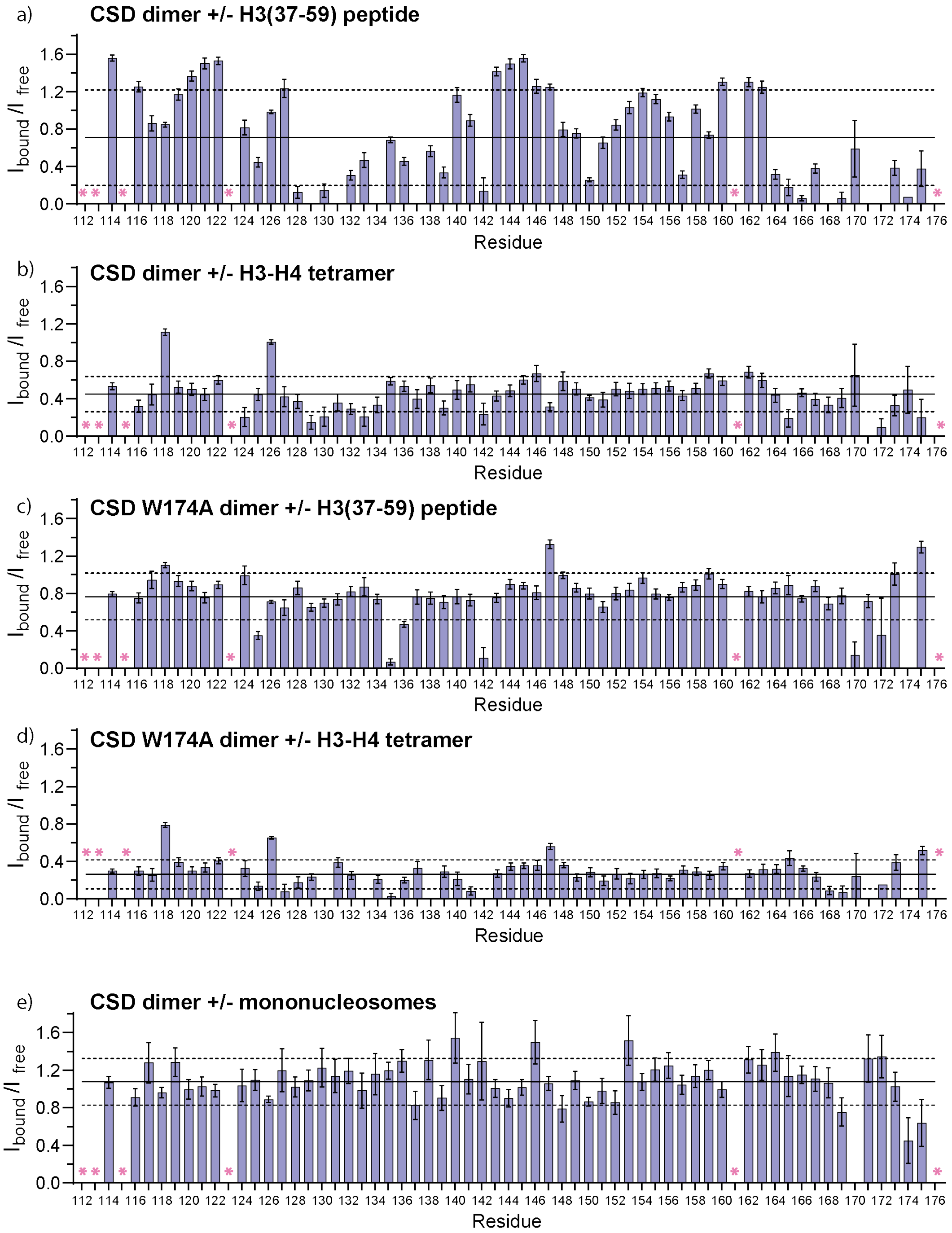

Figure S7. Intensity ratios for the HSQC spectra shown in Fig. 2. a) CSD dimer with and without H3(37-59) peptide in a 1:1 ratio as shown in Fig. 2a. b) CSD dimer with and without H3-H4 tetramer in a 1:0.1 ratio as shown in Fig. 2b. c) CSD W174A dimer with and without H3(37-59) peptide in a 1:1 ratio as shown in Fig. 2c. d) CSD W174A dimer with and without H3-H4 tetramer in a 1:0.1 ratio as shown in Fig. 2d. e) CSD dimer with and without mononucleosomes in a 1:0.8 ratio as shown in Fig. 2h. Asterisks denote residues with missing assignments. The solid line represents the average of the intensity ratios, while the dashed lines represent one standard deviation above and below. Error bars were calculated as summarized in the methods section.

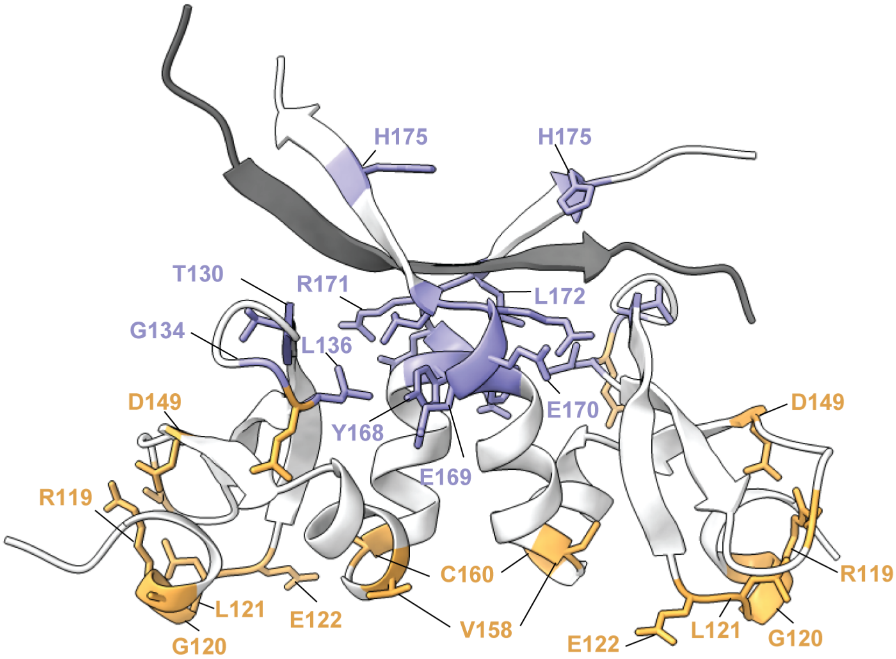

Figure S8. Structure of the CSD dimer with a bound peptide. The structure is from Ref. (14), PDB ID 3Q6S, while the numbering corresponds to the HP1α CSD sequence. Residues that change intensity in the presence of a specific binder (e.g. Fig. 2a) are shown in purple, while residues that do not change their intensity are shown in gold.

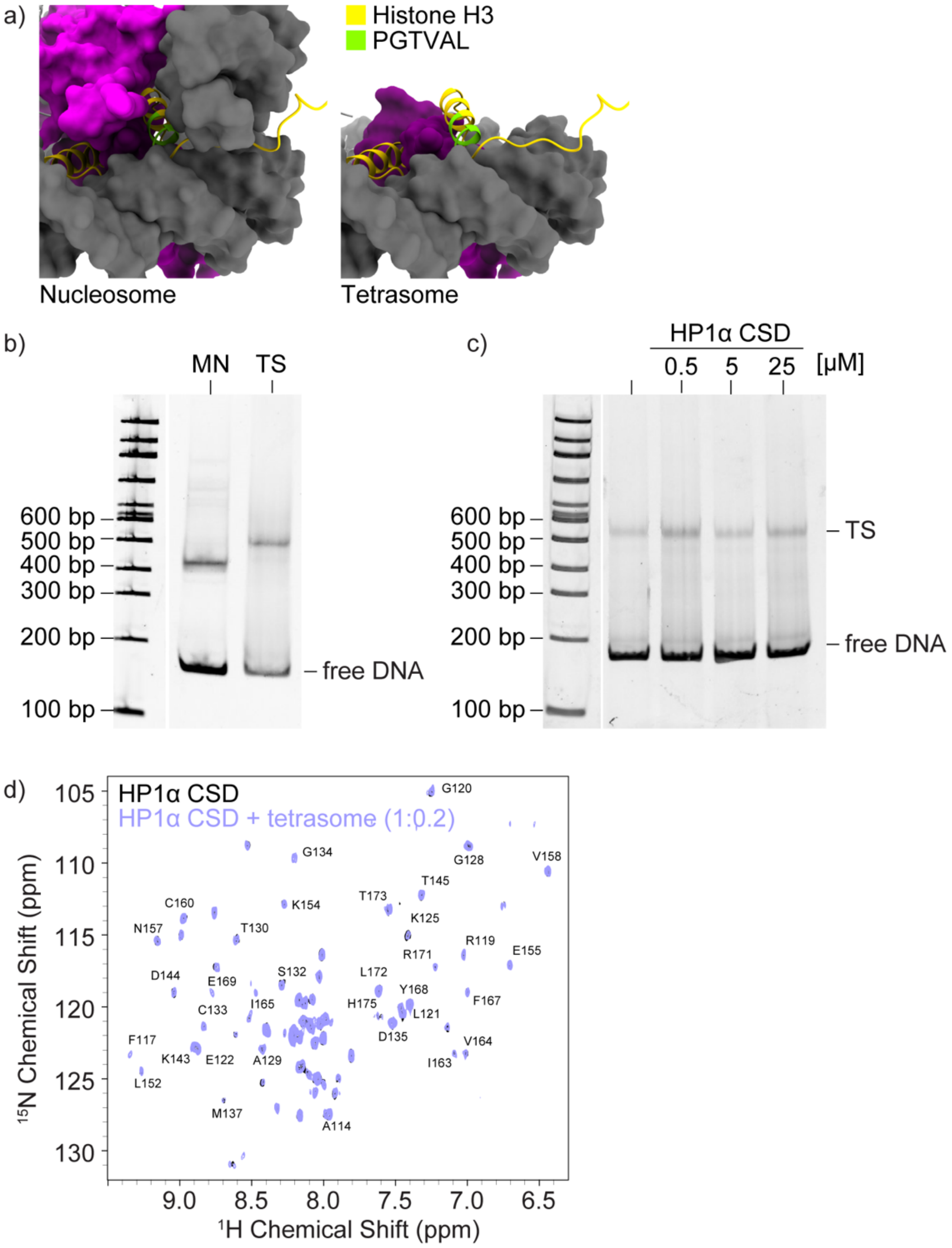

Figure S9. Chromoshadow domain interactions with tetrasomes**.** a) Comparison of H3 exposure with doubly wrapped DNA in a mononucleosome (MN) versus singly wrapped DNA in a tetrasome (TS). The tetrasome image was artificially created based on the nucleosome structure PDB:1KX5, by removing the top DNA strand and H2A/H2B histones. b) Native-PAGE gel comparing the tetrasome (TS) to a mononucleosome (MN). The extended DNA in the TS causes it to run at a higher molecular weight. C) EMSA of TS with varying concentrations of HP1α CSD. D) ^{1}H-^{15}N HSQC of HP1α CSD (black) and HP1α CSD 1:0.2 with TS (purple).

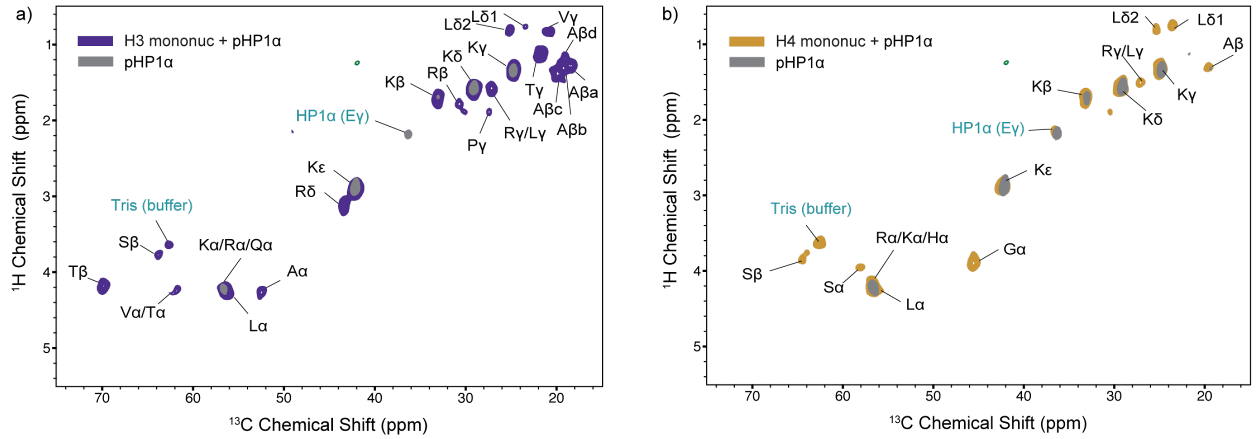

Figure S10. Contribution of natural abundance pHP1α to the ^{1}H-^{13}C INEPT MAS NMR spectra of a) H3-labeled mononucleosomes, and b) H4-labeled mononucleosomes. To estimate the contribution of phase separated natural abundance pHP1α, which is present in large excess in the MAS NMR samples, we took advantage of the Eγ peak, which is only present in pHP1α and not in the H3 or H4 mononucleosome spectra. We, therefore, recorded a spectrum of phase separated pHP1α without mononucleosomes (grey) and scaled the intensity of the spectrum, so that the intensities of the Eγ peaks in the spectra with or without mononucleosomes were equal. After scaling, significant intensity remained for some peaks, e.g. those corresponding to lysine correlations. That intensity was subtracted from the I_{bound} peak intensity before comparing to the I_{free} intensity as shown in Figure 3e,f to represent changes in dynamics in the H3 and H4 tails more accurately.

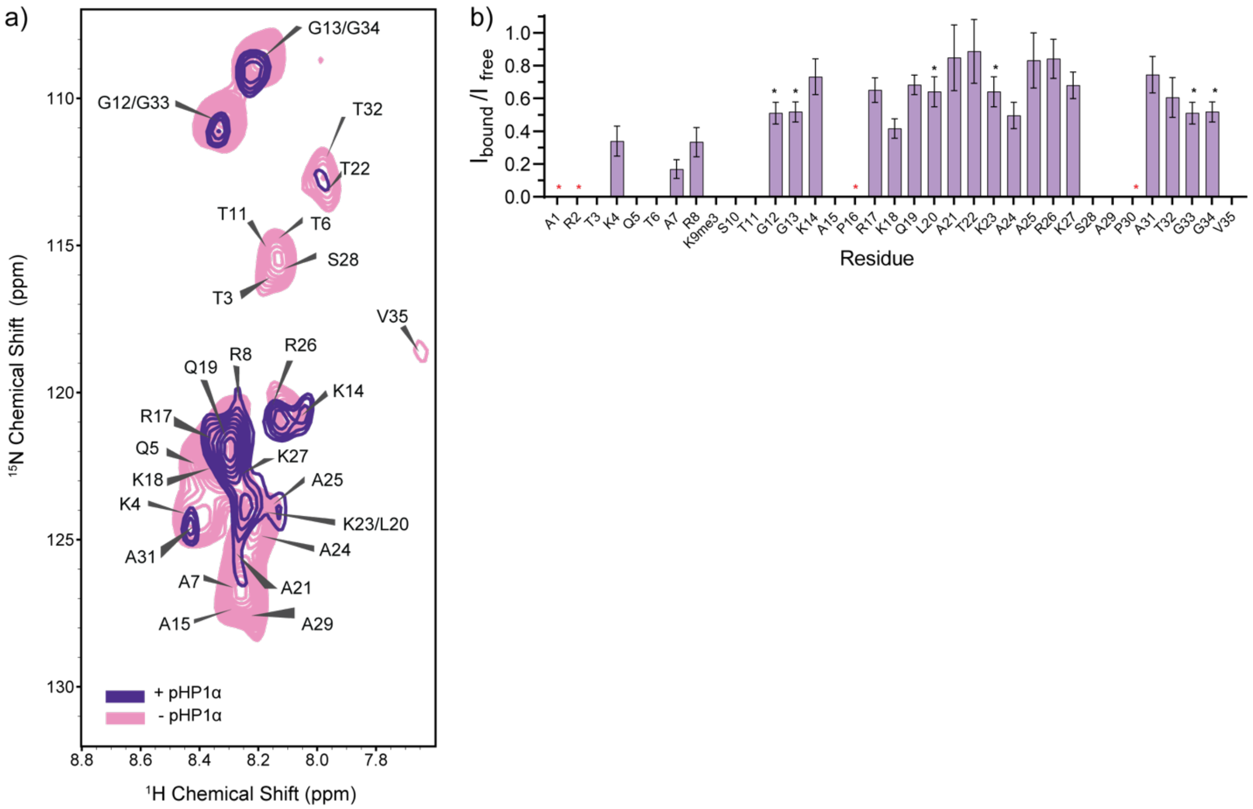

Figure S11. a) 2D ^{1}H-^{15}N INEPT MAS NMR spectra of ^{13}C,^{15}N-H3 labeled nucleosomes in the presence (purple) and absence of phase separated pHP1α (pink). The assignments were transferred from the solution NMR spectra presented in Figure 1. b) Intensity ratios for different residues in the spectra, indicating that residues near the K9Cme3 binding site lose more intensity in the presence of pHP1α. This implies that pHP1a binds nucleosomes in a specific manner in phase separated environments. Black asterisks denote overlapped peaks where analysis for the individual residue could not be performed, i.e. G13/G34, G12/G33, L20/K23, and red asterisks denote residues with missing peaks in the control spectrum. The reported intensity ratios represent positions in the spectrum that match the solution NMR assignments, and only positions with signal-to-noise ratios above 4 were included in the analysis.

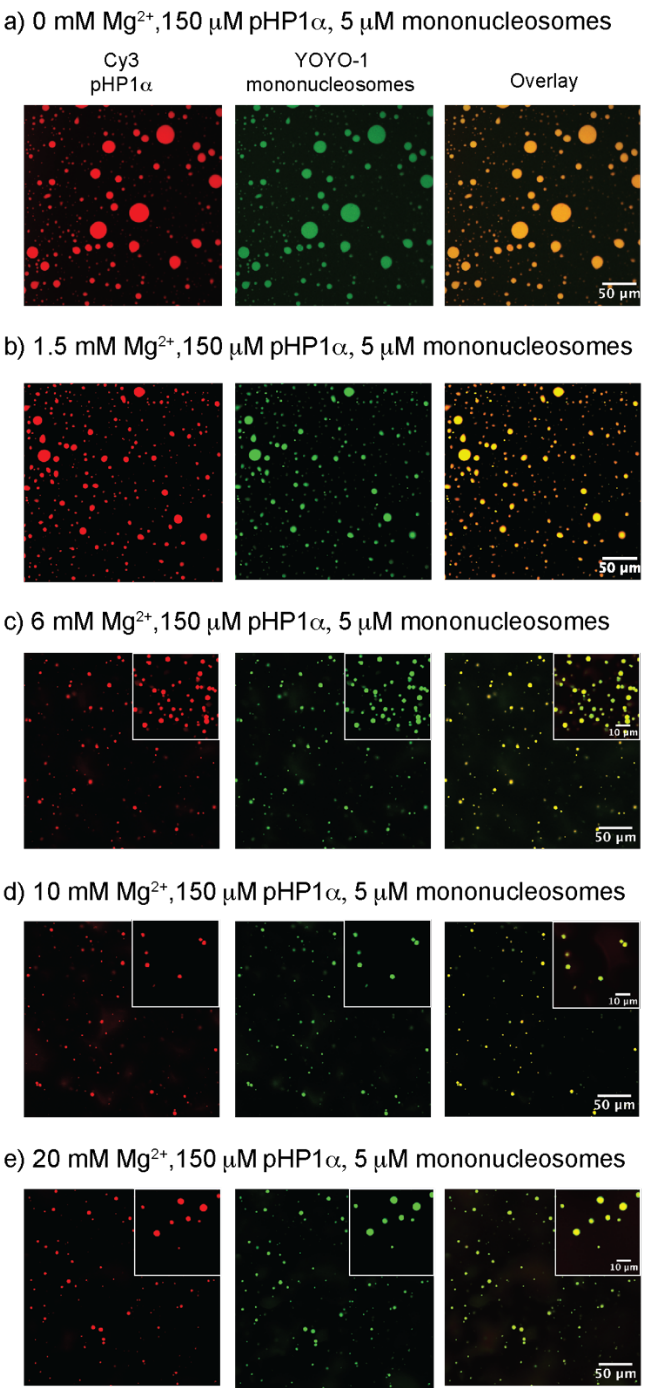

Figure S12. Fluorescence images of samples containing pHP1α and nucleosomes in a 30:1 ratio, similar to the ratio used in the solid-state NMR experiments in the presence of a) 0 mM Mg^{2+}, b) 1.5 mM Mg^{2+}, c) 6 mM Mg^{2+}, d) 10 mM Mg^{2+}, and e) 20 mM Mg^{2+}. All experiments were performed in 10 mM Tris, 0.1 mM EDTA, 10 mM KCl, pH 7.5. Samples were visualized with 5% Cy3-labeled pHP1α and 1 μM YOYO-1 dye, which reports on the location of DNA. The bar corresponds to 50 μm while the insets in c), d), and e) show higher magnification with a bar corresponding to 10 μm.

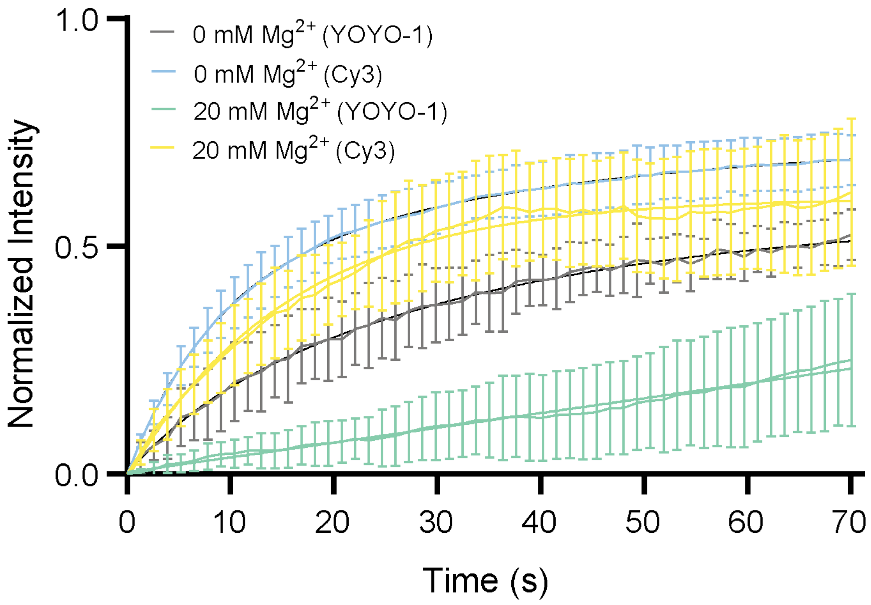

Figure S13. Fluorescence recovery after photobleaching (FRAP) experiments of pHP1α and nucleosome droplets in the presence and absence of Mg^{2+}. All experiments were performed in 10 mM Tris, 0.1 mM EDTA, 10 mM KCl, pH 7.5. Samples were visualized with 5% Cy3-labeled pHP1α and 1 μM YOYO-1 dye, which reports on the location of DNA. pHP1α retains its mobility irrespective of Mg^{2+}, while the recovery of YOYO-1 fluorescence slowed down in the presence of 20 mM Mg^{2+}, which suggests mononucleosome oligomerization.

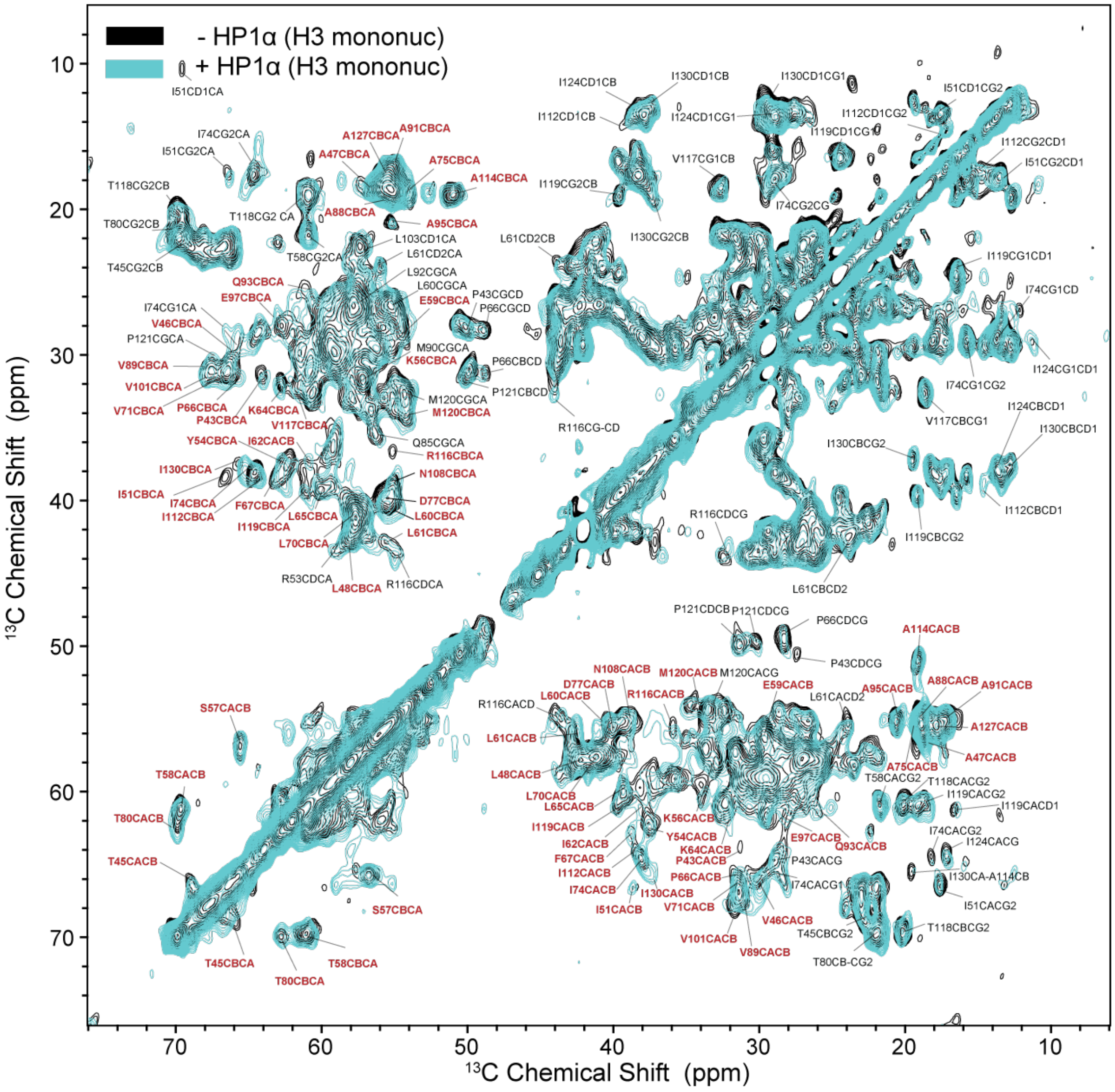

Figure S14. 2D ^{13}C-^{13}C DARR spectra of H3-mononucleosomes in the presence and absence of pHP1α phase separated environments. Spectra were collected at 750 MHz ^{1}H Larmor frequency, 11 kHz MAS spinning frequency, in the presence of 1.5 mM Mg^{2+}. Spectra contain ~ 5 mg of nucleosomes or ~ 0.75 mg of ^{15}N,^{13}C,-labeled H3.

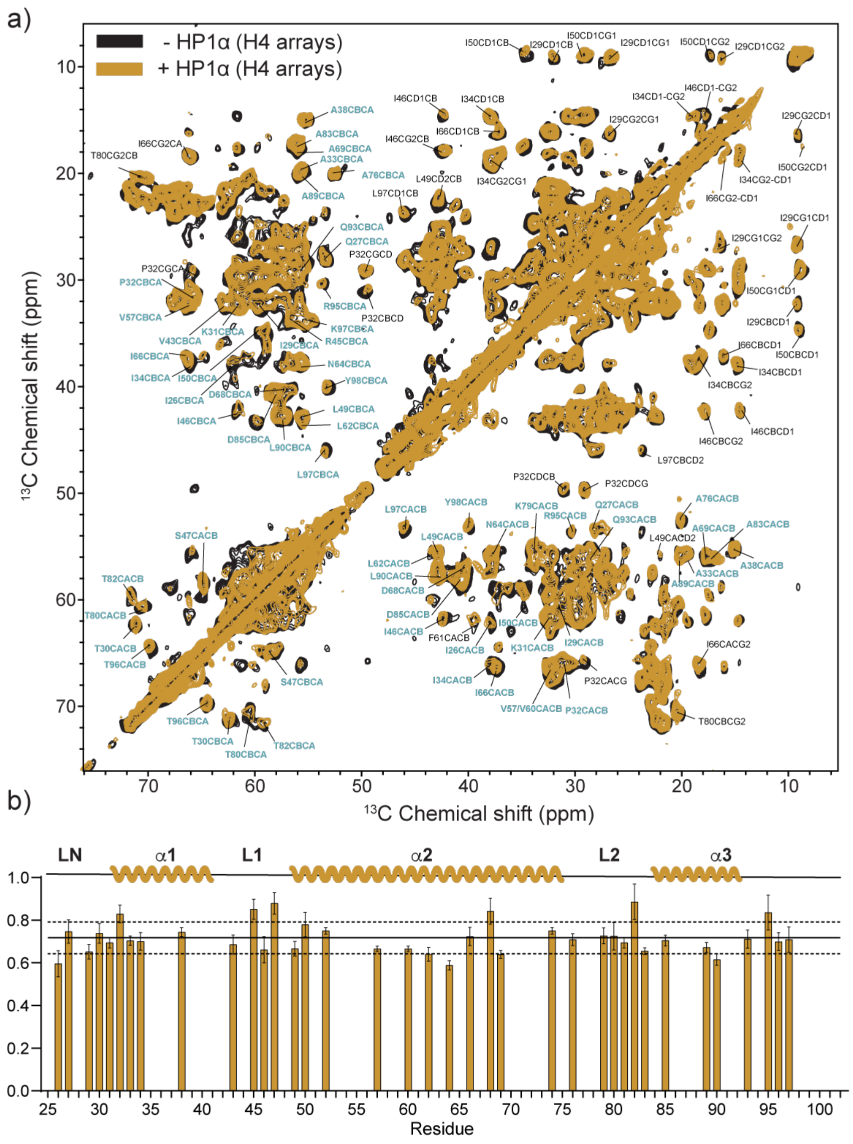

Figure S15. MAS NMR spectroscopy of H4-labeled arrays in pHP1α phase separated environments. a) Comparison of 2D ^{13}C-^{13}C DARR correlations for ^{13}C,^{15}N-H4 labeled 12-mer nucleosome arrays in the presence and absence of pHP1α. Assignments were transferred from Shi *et. al.* (15) b) Analysis of cross-peak intensity for resolved and assigned peaks in the spectra. The data represent the average contribution from Cα-Cβ correlations on both sides of the diagonal for each analyzed residue. The dotted lines represent one standard deviation from the average. Note that the lower average ratio (~0.7) is due to the lower amount of sample in the pHP1α sample (5 mg vs 7 mg of arrays). Spectra were obtained at 750 MHz ^1H Larmor frequency, 11 kHz MAS spinning frequency, in the presence of 6 mM Mg^{2+}.

Supporting Tables

Table S1. Experimental parameters for solution NMR ^{1}H-^{15}N TROSY-HSQC experiments with the CSD domain.

Sample	Temp (K)	Scans	Points/Acq. Time Direct dimension	Points/Acq. Time Indirect dimension	Figure
10 µM ^{15}N CSD dimer + 8 µM H3 mononucleosomes	298	184	2048/90 ms	96/16.4 ms	2h
50 µM ^{15}N CSD dimers + 50 µM H3 peptide	298	24	2048/90 ms	96/16.4 ms	2a
50 µM ^{15}N CSD dimers + 5 µM tetramer	298	24	2048/90 ms	96/16.4 ms	2b
50 µM ^{15}N CSD W174A dimers + 50 µM H3 peptide	298	24	2048/90 ms	96/16.4 ms	2c
50 µM ^{15}N CSD W174A dimers + 5 µM tetramer	298	24	2048/90 ms	96/16.4 ms	2d
25 µM ^{15}N CSD dimers + 5 µM tetrasome	293	32	2048/90 ms	128/21.9 ms	S9d

Table S2. Experimental parameters for MAS NMR experiments with H3-labeled mononucleosomes as presented in Figure 4.

Experiment	H3 control		H3 + pHP1α	
Nucleosome amount	10	mg	10	mg
pHP1α added	0	mg	15	mg
**CP**				
Number of scans	512	scans	512	scans
Recycle delay	5	s	5	s
CP contact time	0.5	ms	0.5	ms
Decoupling	SPINAL64		SPINAL64	
Decoupling power	83	kHz	83	kHz
Acquisition time	9	ms	9	ms
**INEPT**				
Number of scans	512	scans	512	scans
Recycle delay	4	s	4	s
^{13}C 90	5	us	5	us
^{1}H 90	3	us	3	us
Refocusing parameter	140	Hz	140	Hz
Decoupling	SPINAL64		SPINAL64	
Decoupling power	83	kHz	83	kHz
Acquisition time	9	ms	9	ms
**^{1}H-^{13}C INEPT**				
Number of scans	64	scans	64	scans
Recycle delay	4	s	4	s
^{13}C 90	5	us	5	us
^{1}H 90	3	us	3	us
Refocusing parameter	140	Hz	140	Hz
Decoupling	swftppm		swftppm	
Decoupling power	83	kHz	83	kHz
t1 acquisition	33.3	ms	33.3	ms
t1 points	500		500	
**^{13}C-^{13}C DARR**				
Number of scans	144	scans	144	scans
Recycle delay	4	s	4	s
CP contact time	1.2	ms	1.2	ms
Decoupling	SPINAL64		SPINAL64	
Decoupling power	83	kHz	83	kHz
1H power during DARR	15	kHz	15	kHz
DARR mixing	20	ms	20	ms
t1 acquisition	3.2	ms	3.2	ms
t1 points	256		256	

Table S3. Experimental parameters for MAS NMR experiments with H4-labeled mononucleosomes and array samples as presented in Figure 5 and Figure S15.

**Experiment**	**H4 (mononuc) control**		**H4 (mononuc)+ pHP1α**	
Nucleosome amount	10	mg	10	mg
pHP1α added	0	mg	20	mg
**^{1}H-^{13}C INEPT**				
Number of scans	64	scans	64	scans
Recycle delay	4	s	4	s
^{13}C 90	5	us	5	us
^{1}H 90	3	us	3	us
Refocusing parameter	140	Hz	140	Hz
Decoupling	swftppm		swftppm	
Decoupling power	83	kHz	83	kHz
t1 acquisition	33.3	ms	33.3	ms
t1 points	500		500	
**^{13}C-^{13}C DARR**				
Number of scans	736	scans	736	scans
Recycle delay	4	s	4	s
CP contact time	1.2	ms	1.2	ms
Decoupling	SPINAL64		SPINAL64	
Decoupling power	83	kHz	83	kHz
H power during DARR	11.1	kHz	11.1	kHz
DARR mixing	20	ms	20	ms
t1 acquisition	3.2	ms	3.2	ms
t1 points	256		256	
**Experiment**	**H4 (arrays) control**		**H4 (arrays)+ pHP1α**	
Nucleosome amount	7	mg	5	mg
pHP1α added	0	mg	15	mg
**^{13}C-^{13}C DARR**				
Number of scans	368	scans	368	scans
Recycle delay	4	s	4	s
CP contact time	1.2	ms	1.2	ms
Decoupling	SPINAL64		SPINAL64	
Decoupling power	83	kHz	83	kHz
^{1}H power during DARR	11.1	kHz	11.1	kHz
DARR mixing	20	ms	20	ms
t1 acquisition	3.2	ms	3.2	ms
t1 points	256		256	
